# Precious-Metal-Free Mo-MXene Catalyst Enabling Facile Ammonia Synthesis Via Dual Sites Bridged by H-Spillover

**DOI:** 10.1021/jacs.4c03998

**Published:** 2024-08-12

**Authors:** Yanliang Zhou, Lili Liang, Congying Wang, Fuxiang Sun, Lirong Zheng, Haifeng Qi, Bin Wang, Xiuyun Wang, Chak-tong Au, Junjie Wang, Lilong Jiang, Hideo Hosono

**Affiliations:** † National Engineering Research Center of Chemical Fertilizer Catalyst, Fuzhou University, Fuzhou 350002, China; ‡ State Key Laboratory of Solidification Processing, School of Materials Science and Engineering, Northwestern Polytechnical University, Xi’an 710072, China; § Institute of High Energy Physics, Chinese Academy of Sciences, Beijing 100049, China; ∥ Leibniz-Institut für Katalyse e.V., Rostock 18059, Germany; ⊥ Sinopec Beijing Research Institute of Chemical Industry, Beijing 100013, China; # MDX Research Center for Element Strategy, Tokyo Institute of Technology, Yokohama, Kanagawa 226-8503, Japan

## Abstract

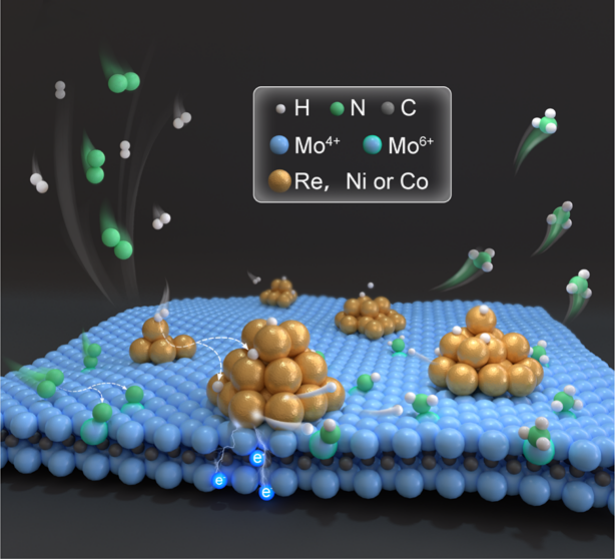

To date, NH_3_ synthesis under mild conditions is largely confined to precious Ru catalysts, while nonprecious metal (NPM) catalysts are confronted with the challenge of low catalytic activity due to the inverse relationship between the N_2_ dissociation barrier and NH_*x*_ (*x* = 1–3) desorption energy. Herein, we demonstrate NPM (Co, Ni, and Re)-mediated Mo_2_CT_*x*_ MXene (where T_*x*_ denotes the OH group) to achieve efficient NH_3_ synthesis under mild conditions. In particular, the NH_3_ synthesis rate over Re/Mo_2_CT_*x*_ and Ni/Mo_2_CT_*x*_ can reach 22.4 and 21.5 mmol g^–1^ h^–1^ at 400 °C and 1 MPa, respectively, higher than that of NPM-based catalysts and Cs–Ru/MgO ever reported. Experimental and theoretical studies reveal that Mo^4+^ over Mo_2_CT_*x*_ has a strong ability for N_2_ activation; thus, the rate-determining step is shifted from conventional N_2_ dissociation to NH_2_* formation. NPM is mainly responsible for H_2_ activation, and the high reactivity of spillover hydrogen and electron transfer from NPM to the N-rich Mo_2_CT_*x*_ surface can efficiently facilitate nitrogen hydrogenation and the subsequent desorption of NH_3_. With the synergistic effect of the dual active sites bridged by H-spillover, the NPM-mediated Mo_2_CT_*x*_ catalysts circumvent the major obstacle, making NH_3_ synthesis under mild conditions efficient.

## Introduction

Ammonia (NH_3_) is a fundamental building block in the chemical industry and is considered a promising carrier of carbon-free hydrogen energy.^1−3^ In the synthesis of NH_3_ via the Haber–Bosch (HB) process, 1–2% of global energy is consumed for NH_3_ synthesis, including hydrogen formation from natural gas (mainly CH_4_), and 1.5–1.9 tons of carbon dioxide (CO_2_) emission per ton of NH_3_ comes from the former H_2_ production process.^4^ To reduce fossil fuel dependence and carbon footprint, the synthesis of NH_3_ following the electrolysis-driven Haber–Bosch (*e*HB) protocol of “renewable energy → electrolytic H_2_ production → NH_3_ synthesis” is meaningful.^5,6^ The major obstacle to the *e*HB process is that the output pressure of H_2_ generated from pressurized water electrolysis (mostly in the range of 1.0–3.2 MPa) does not match the adopted conditions over traditional Fe-based catalysts for NH_3_ synthesis (15–30 MPa), and installation for pressure ramping is cumbersome and expensive.^7,8^ To make *e*HB cost-effective, it is important to develop catalysts that can efficiently operate under milder conditions (≤400 °C and 3 MPa) for NH_3_ synthesis.

The major obstacle to NH_3_ synthesis under mild conditions lies in the inverse relationship between the dissociation barrier of N_2_ and the desorption energy of NH_*x*_ (*x* = 1–3) species on transition metals (TMs).^9,10^ Therefore, the NH_3_ synthesis rates over the TMs exhibit a volcano curve relationship with the nitrogen (N) adsorption energy on the TMs.^9^ According to this volcano curve, Ru with moderate N adsorption energy is located near the peak of catalytic activity, while the nonprecious metals (NPMs) on either side of the plot cannot convert N_2_ to NH_3_ efficiently due to too strong or weak N adsorption, thus resulting in a higher NH_*x*_ desorption energy or higher N_2_ dissociation barrier, respectively.^11^ Much effort has been devoted to the development of NPM-based catalysts that can overcome these obstacles.^12−14^ Recently, TMs/nitrides have been reported to efficiently catalyze NH_3_ synthesis due to their separated sites for H_2_ adsorption over TMs and facile N_2_ activation over nitride ion vacancies.^15,16^ Although this strategy is highly effective, it is highly dependent on metal nitrides with low nitrogen vacancy formation energy to promote N_2_ activation. Unfortunately, these nitrides are usually highly sensitive to air and moisture, which limits their practical applications. Besides, the reaction of activated reactants over different sites is supposed to take place at the interface of dual sites, which not only reduces the effective active sites for NH_3_ formation but also raises the difficulty in precisely regulating the spatial position of the dual sites. This largely limits the potential application of the dual-site strategy for NH_3_ synthesis over other catalytic materials. Hydrogen spillover (H-spillover) can be associated with different active sites via the transfer of activated H species, which are highly active to hydrogenate adsorbed reactants such as N_2_ and NH_*x*_. In addition, H-spillover, which involves the transfer of hydrogen protons and electrons, can facilitate electron transport at different sites,^17,18^ thus altering the ad/desorption properties of products over the catalyst surface. Taking these characteristics of dual sites and H-spillover into consideration, we considered that the integration of H-spillover with a dual-site strategy may enable NPM-based catalysts to simultaneously realize the efficient activation of N_2_ and subsequent facile formation of NH_*x*_/desorption of NH_3_.

Recently, theoretical calculations have suggested that two-dimensional (2D) MXenes (denoted as M_*n*+1_X*_n_*T_*x*_ (*n* = 1–3), where the transition metal (M) is intercalated into a layer of carbon or nitride (X = C or N), and T_*x*_ denotes the surface functional group) are potential catalysts for NH_3_ synthesis due to their flexible composition and adjustable active centers.^19−21^ In addition, due to the unique ion transport properties of 2D MXenes, the surface functional groups are exchangeable and H-spillover occurs on the MXene surfaces.^22,23^ Although N_2_ activation over some MXenes is feasible, as demonstrated by numerous theoretical investigations,^24−26^ there are still no experimental applications of MXene in thermocatalytic NH_3_ synthesis because individual MXenes cannot circumvent the major obstacle of NH_3_ synthesis, as mentioned above, to achieve the desired performance. Considering the positive factors of dual sites and H-spillover on 2D MXene materials, the combination of MXenes with a second active metal for the formation of bifunctional catalysts with H-spillover assistance is a possible strategy for extracting the unique features of MXene for NH_3_ synthesis.

Herein, we report for the first time the mediation of partially defunctionalized Mo_2_CT_*x*_ by NPM, including Co, Ni, and Re to realize efficient NH_3_ synthesis under mild conditions using the dual-site strategy and H-spillover effect. Therefore, the NH_3_ synthesis rates over Re/Mo_2_CT_*x*_ and Ni/Mo_2_CT_*x*_ can reach up to 22.4 and 21.5 mmol g^–1^ h^–1^ at 400 °C and 1 MPa, respectively, which is higher than that of Mo- or Re-based catalysts reported and also comparable to that of Ru-based catalysts. Various characterization and density functional theory (DFT) calculations reveal that Mo^4+^ species over defunctionalized Mo_2_CT_*x*_ serve as active sites for N_2_ activation, while the NPM sites are mainly responsible for H_2_ activation, and the rate-determining step (RDS) is the formation of NH_2_*. The spillover of H* species from NPM to Mo_2_CT_*x*_ can promote the hydrogenation of dissociated N species to form NH_*x*_ (*x* = 1–3) intermediates on the Mo active sites. Meanwhile, the charge transfer from NPM to Mo_2_CT_*x*_ could accelerate the desorption of NH_3_, thereby enabling NH_3_ synthesis with high efficiency under mild conditions.

## Results

### Structure Properties of Mo_2_CT_*x*_ MXene

**Figure 1:**
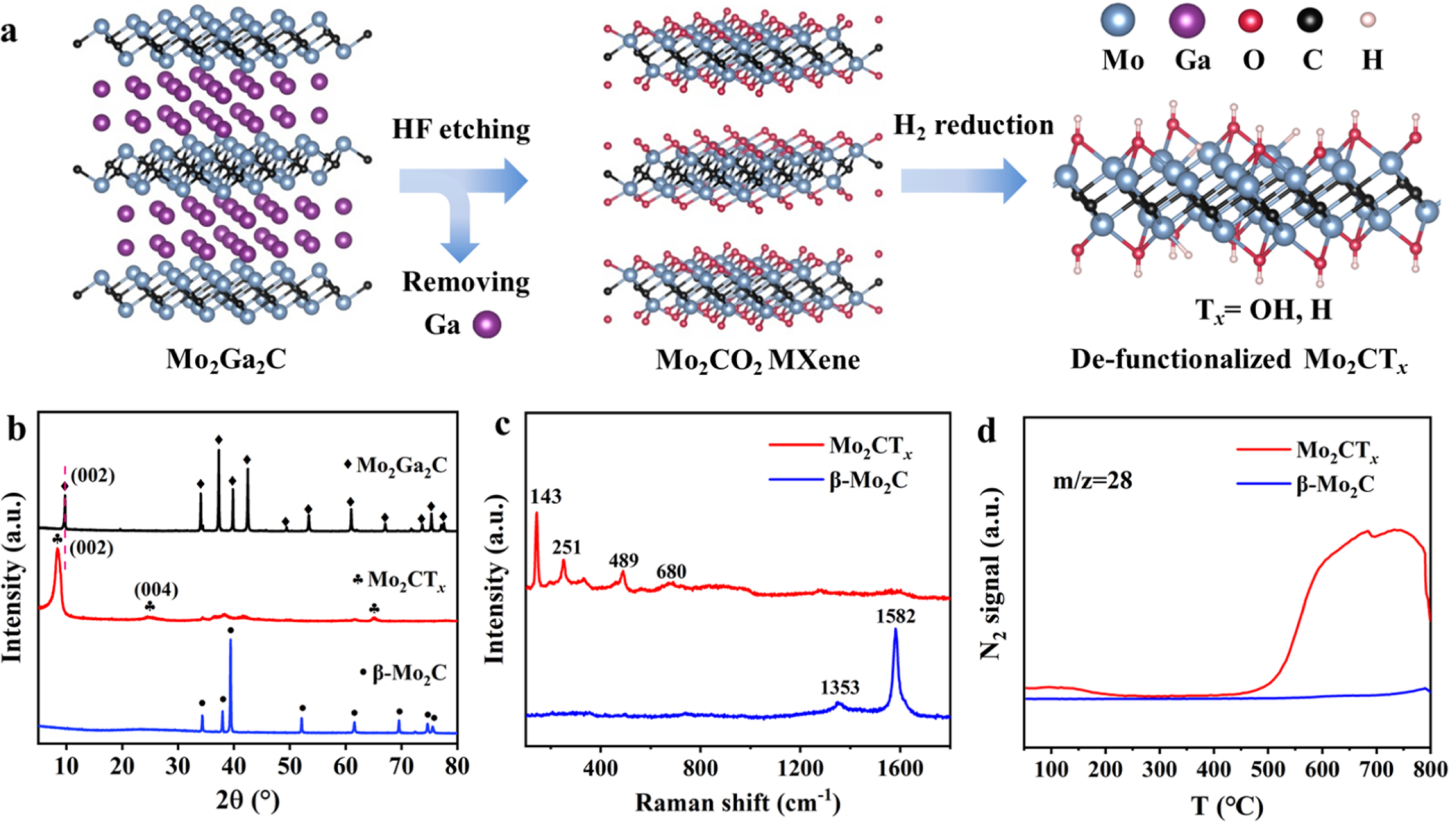
Synthesis and characterization of the Mo_2_CT_*x*_ catalyst. (a) Schematic showing the synthesis process of 2D Mo_2_CT_*x*_ MXene. (b) XRD patterns of Mo_2_CT_*x*_, Mo_2_Ga_2_C, and β-Mo_2_C. (c) Raman spectra of Mo_2_CT_*x*_ and Mo_2_C. (d) N_2_-TPD profiles of Mo_2_CT_*x*_ and β-Mo_2_C were recorded using online MS.

The schematic of the synthesis of Mo_2_CT_*x*_ MXene is shown in Figure 1a. The Mo_2_Ga_2_C precursor was etched with hydrofluoric acid (HF) and then washed with an alkali solution to remove Ga and F species (more details are provided in the Experimental Section). Energy-dispersive X-ray spectroscopy (EDX, Figure S1) results reveal that the residual F (<0.01 wt %) and Ga (0.22 wt %) contents are very low, and Mo, C, and O are uniformly dispersed throughout Mo_2_CT_*x*_. The inductively coupled plasma-atomic emission spectroscopy (ICP-AES) results show that the residual G content is around 0.2 wt %, indicating that the Ga is almost fully etched from Mo_2_Ga_2_C. X-ray diffraction (XRD) analysis reveals the characteristic (002) and (004) peaks of Mo_2_CT_*x*_ (Figure 1b), and the position of the (002) peak decreases from 9.75° to 8.39° compared to Mo_2_Ga_2_C. This result indicates an enlarged interplanar spacing upon the removal of the Ga layer and the successful synthesis of Mo_2_CT_*x*_. As determined by scanning electron microscopy (SEM, Figure S2), Mo_2_CT_*x*_ presents an accordion-like morphology, consisting of multilayer 2D MXene. In the Raman spectra (Figure 1c) of Mo_2_CT_*x*_, the peaks at 143 and 251 cm^–1^ are attributed to the symmetric and antisymmetric vibrational C–Mo–C modes, while the peaks at ∼489 and 680 cm^–1^ are ascribed to analogous modes of O–Mo–O.^27^ These results indicate that the surface of Mo_2_CT_*x*_ is mainly terminated by oxygen species, which is in accordance with the Mo_2_CO_2_ MXene structure, as reported previously.^20,27^ For comparison, β-Mo_2_C was chosen as a reference. Based on the results of XRD (Figure 1b) and nitrogen physical adsorption analyses, despite the similarity in Mo-to-C composition, the exposed crystal planes and specific surface areas between Mo_2_CT_*x*_ (16 m^2^ g^–1^) and β-Mo_2_C (7 m^2^ g^–1^) are different.

H_2_-temperature-programmed reduction (H_2_-TPR, Figure S3) confirms that the surface oxygen species of Mo_2_CT_*x*_ could be reduced to generate H_2_O with an increase of the reduction temperature.^28^ Quantitative analysis of hydrogen consumption suggested that 68% of the surface oxygen species on Mo_2_CT_*x*_ were eliminated, resulting in the formation of partially defunctionalized Mo_2_CT_*x*_, i.e., a part of the oxygen functional groups of Mo_2_CT_*x*_ was removed. After reduction, the dynamic behavior of the H species was studied by H_2_-temperature-programmed desorption using an online mass spectrometer (H_2_-TPD-MS, Figure S4). Two obvious desorption peaks were observed at 140 and 418 °C. Since each of the peak temperatures distinctly differs from that due to dehydration, as shown in Figure S3, we tentatively assigned it to H_2_ desorption evolved from the surface Mo–H and Mo–OH species, respectively.^29^ These results demonstrate that the oxygen functional groups of Mo_2_CT_*x*_ can be removed or partially transferred into H or OH groups under a reducing atmosphere (Figure 1a). In the N_2_-TPD-MS investigation (Figure 1d), an intense and broad desorption peak was observed over Mo_2_CT_*x*_, while no obvious peak was discerned over β-Mo_2_C, indicating that Mo_2_CT_*x*_ was conducive to the adsorption of N_2_. These results demonstrate that partially defunctionalized 2D Mo_2_CT_*x*_ can provide a highly active platform for N_2_ and H_2_ activation and it could be a potential catalyst for NH_3_ synthesis.

### Catalytic Performance for the NH_3_ Synthesis Reaction

**Figure 2:**
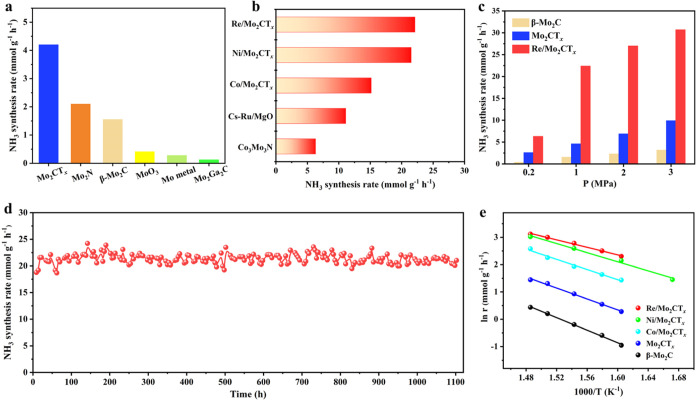
Catalytic performance for NH_3_ synthesis. NH_3_ synthesis rates over (a) different Mo-based catalysts and (b) NPM-mediated Mo_2_CT_*x*_ samples as well as reference catalysts at 400 °C and 1 MPa. (c) NH_3_ synthesis rate versus reaction pressure for different catalysts at 400 °C. (d) Long-term stability test over Re/Mo_2_CT_*x*_ at 400 °C and 1 MPa. (e) Arrhenius plots of NPMs/Mo_2_CT_*x*_, Mo_2_CT_*x*_, and β-Mo_2_C.

The NH_3_ synthesis performance of the as-prepared catalysts was evaluated in a 25%N_2_–75%H_2_ feed at weight hourly space velocity (WHSV) of 60 000 mL g^–1^ h^–1^. Given that there is no obvious weight loss of Mo_2_CT_*x*_ in the thermogravimetric analysis (Figure S5) and that its structure can remain stable below 600 °C,^28,30^ fresh Mo_2_CT_*x*_ was reduced at 500 °C for 2 h to obtain partially defunctionalized Mo_2_CT_*x*_ before measurements. As shown in Figure 2a, the NH_3_ synthesis rate over reduced Mo_2_CT_*x*_ is 4.2 mmol g^–1^ h^–1^ at 400 °C and 1 MPa, which is around 2.8 times that of β-Mo_2_C (1.5 mmol g^–1^ h^–1^). To highlight the outstanding performance of Mo_2_CT_*x*_, other Mo-based catalysts, including Mo_2_N, MoO_3_, metallic Mo, and Mo_2_Ga_2_C, were prepared and evaluated for comparison, the crystal phases of which were confirmed by XRD analysis (Figure S6). Catalytic performance tests (Figure 2a) show that the NH_3_ synthesis rate over Mo_2_CT_*x*_ is around 2-, 10-, 15-, and 36-fold higher than those over Mo_2_N, MoO_3_, metallic Mo, and Mo_2_Ga_2_C, respectively. Note that the NH_3_ synthesis rate over metallic Mo was only 0.28 mmol g^–1^ h^–1^, which is consistent with the volcano curve of NH_3_ synthesis that suggests that Mo metal is not ideal for NH_3_ synthesis due to strong adsorption of N species.

In view of the unique properties of Mo_2_CT_*x*_, the combination of Mo_2_CT_*x*_ and NPM is expected to be promising for NH_3_ synthesis. Typical NPMs, including Co, Ni, and Re were loaded onto Mo_2_CT_*x*_ to obtain the Co/Mo_2_CT_*x*_, Ni/Mo_2_CT_*x*_, and Re/Mo_2_CT_*x*_ catalysts, respectively. Obviously, there is a significant and common increase in the NH_3_ synthesis rate after the loading of NPM on Mo_2_CT_*x*_ (Figure 2b), irrespective of whether it is an early (Re) or late (Co and Ni) TMs. The NH_3_ synthesis rates over Co/Mo_2_CT_*x*_, Ni/Mo_2_CT_*x*_, and Re/Mo_2_CT_*x*_ are 15.2, 21.5, and 22.4 mmol g^–1^ h^–1^ at 400 °C and 1 MPa, respectively. In terms of Re/Mo_2_CT_*x*_, the outlet concentration of NH_3_ and conversion of N_2_ are around 0.93% and 1.86%, respectively. Notably, the NH_3_ synthesis rate of Re/Mo_2_CT_*x*_ is the highest ever reported among nonprecious Mo- or Re-based catalysts (Table S1) and even outperforms typical noble Ru-based catalysts. For example, Re/Mo_2_CT_*x*_ is significantly higher than the benchmark Co_3_Mo_3_N (6.3 mmol g^–1^ h^–1^) and noble metal catalyst Cs-5%Ru/MgO (11.1 mmol g^–1^ h^–1^) in performance (for more details on the preparation of Co_3_Mo_3_N and Cs-5%Ru/MgO, see Experimental Section, and their structures are provided in Figure S7).^31^ Additionally, the NH_3_ synthesis rate is strongly dependent on NPM loading. For example, the NH_3_ synthesis rate reaches a maximum of 10 wt % Re, 20 wt % Ni, and 10 wt % Co, depending on the nature of the NPM (Figures S8–S10). For a fair comparison, 20 wt % Ni/Mo_2_CT_*x*_ was evaluated under the conditions adopted as Ni/LaN (Table S1).^15^ The catalytic NH_3_ synthesis rate over Ni/Mo_2_CT_*x*_ is 16.3 mmol g^–1^ h^–1^ at 400 °C and 0.9 MPa with WHSV of 36 000 mL g^–1^ h^–1^, which is higher than that of 15.5 mmol g^–1^ h^–1^ over Ni/LaN,^15^ indicating the superior NH_3_ synthesis performance over Ni/Mo_2_CT_*x*_. Note that the loading of the NPM on Mo_2_CT_*x*_ results in a high NH_3_ synthesis rate, reflecting that the catalytic performances are not NPM-dependent as expected from the volcano plot.^32^

The effects of reaction temperature and pressure on the NH_3_ synthesis rate over the as-prepared catalysts were investigated. With an increase of reaction pressure from 0.2 to 3 MPa, the NH_3_ synthesis rate over Re/Mo_2_CT_*x*_ gradually increased from 6.3 to 30.7 mmol g^–1^ h^–1^ (Figure 2c). In addition, the NH_3_ synthesis rate of Re/Mo_2_CT_*x*_ reaches a maximum of 33.0 mmol g^–1^ h^–1^ at 475 °C and 1 MPa with an increase of the reaction temperature, which is consistently higher than that over Mo_2_CT_*x*_ and β-Mo_2_C under the adopted conditions (Figure S11). Catalyst stability is a critical requirement in practical applications. The long-term stability of Re/Mo_2_CT_*x*_ was evaluated at 400 °C and 1 MPa (Figure 2d). The NH_3_ synthesis rate of Re/Mo_2_CT_*x*_ remained stable at ∼22 mmol g^–1^ h^–1^, showing no obvious deactivation in the time-on-stream of 1100 h. The XRD results show that the diffraction peaks of the used Re/Mo_2_CT_*x*_ remain unchanged compared to those of the fresh samples (Figure S12). During NH_3_ synthesis at 400 °C and 1 MPa, the CH_4_ concentration at the outlet was negligibly low (Figure S13), indicating that the carbon entities of Re/Mo_2_CT_*x*_ were highly stable under the adopted conditions. Overall, the superior catalytic performance and long-term operational stability of Re/Mo_2_CT_*x*_ demonstrate its high potential for industrial applications.

### Kinetics Studies

To gain insight into the reaction mechanism of NH_3_ synthesis over the as-prepared catalysts, kinetic analysis was performed. As derived from the Arrhenius plots, the apparent activation energy (*E*_a_) for NH_3_ synthesis over Mo_2_CT_*x*_ is 83 kJ mol^–1^ (Figure 2e), which is lower than that over β-Mo_2_C (96 kJ mol^–1^). After the loading of NPMs on Mo_2_CT_*x*_, the *E*_*a*_ values significantly decreased to 57–74 kJ mol^–1^. Such low *E*_a_ values are comparable to those of Ru-loaded electride and TM-LiH catalysts,^9,33^ suggesting facile N_2_ activation over NPMs/Mo_2_CT_*x*_. Moreover, the high *E*_a_ and large N_2_ reaction order (1.70) of Mo_2_C suggest that the dissociation of N_2_ is kinetically slow (Table 1), which is similar to the conventional Fe- or Ru-based catalysts where the dissociation of N≡N triple bond is regarded as the RDS.^34^ More impressively, the N_2_ reaction orders over Mo_2_CT_*x*_ and NPMs/Mo_2_CT_*x*_ (0.54–0.70) are much lower compared to that over β-Mo_2_C, indicating that Mo_2_CT_*x*_ and NPMs/Mo_2_CT_*x*_ have a higher ability for N_2_ adsorption and activation. The N adatom densities on Mo_2_CT_*x*_ and NPMs/Mo_2_CT_*x*_ are dense, and the NH_3_ synthesis rate is not constrained by the number of dissociated N atoms.^33,35^ It was hence deduced that the RDS of NH_3_ synthesis over Mo_2_CT_*x*_ and NPMs/Mo_2_CT_*x*_ may shift from the direct dissociation of the N≡N triple bond to the formation of N–H bonds (*vide infra*). Moreover, the H_2_ reaction orders of NPMs/Mo_2_CT_*x*_ catalysts are in the range of 0.6–2.1, indicating that the hydrogen poisoning phenomenon over NPM/Mo_2_CT_*x*_ caused by competitive adsorption on active sites can be excluded, notably differing from conventional Ru-based catalysts.^36^ Meanwhile, the reaction orders of NH_3_ over Mo_2_CT_*x*_ and β-Mo_2_C are −1.63 and −2.22, respectively, implying that NH_3_ binds strongly and populates these two catalysts. Interestingly, the reaction orders upon loading NPM_*x*_ are relatively more positive (i.e., – 0.92 for Re/Mo_2_CT_*x*_) compared to that over Mo_2_CT_*x*_. These results demonstrate that the loading of NPMs on Mo_2_CT_*x*_ is beneficial for N_2_ activation and NH_3_ desorption from the catalyst surface.

**Table 1:** Comparison of Kinetic Parameters of As-Prepared Catalysts

catalyst	*E*_a_ (kJ mol^–1^)	α (N_2_)	β (H_2_)	γ (NH_3_)
Re/Mo_2_CT_*x*_	57	0.54	0.69	–0.92
Ni/Mo_2_CT_*x*_	65	0.68	0.63	–1.10
Co/Mo_2_CT_*x*_	74	0.64	0.91	–1.17
Mo_2_CT_*x*_	83	0.70	1.24	–1.63
β-Mo_2_C	96	1.70	2.07	–2.22

### Structural Identification of Re/Mo_2_CT_*x*_

**Figure 3:**
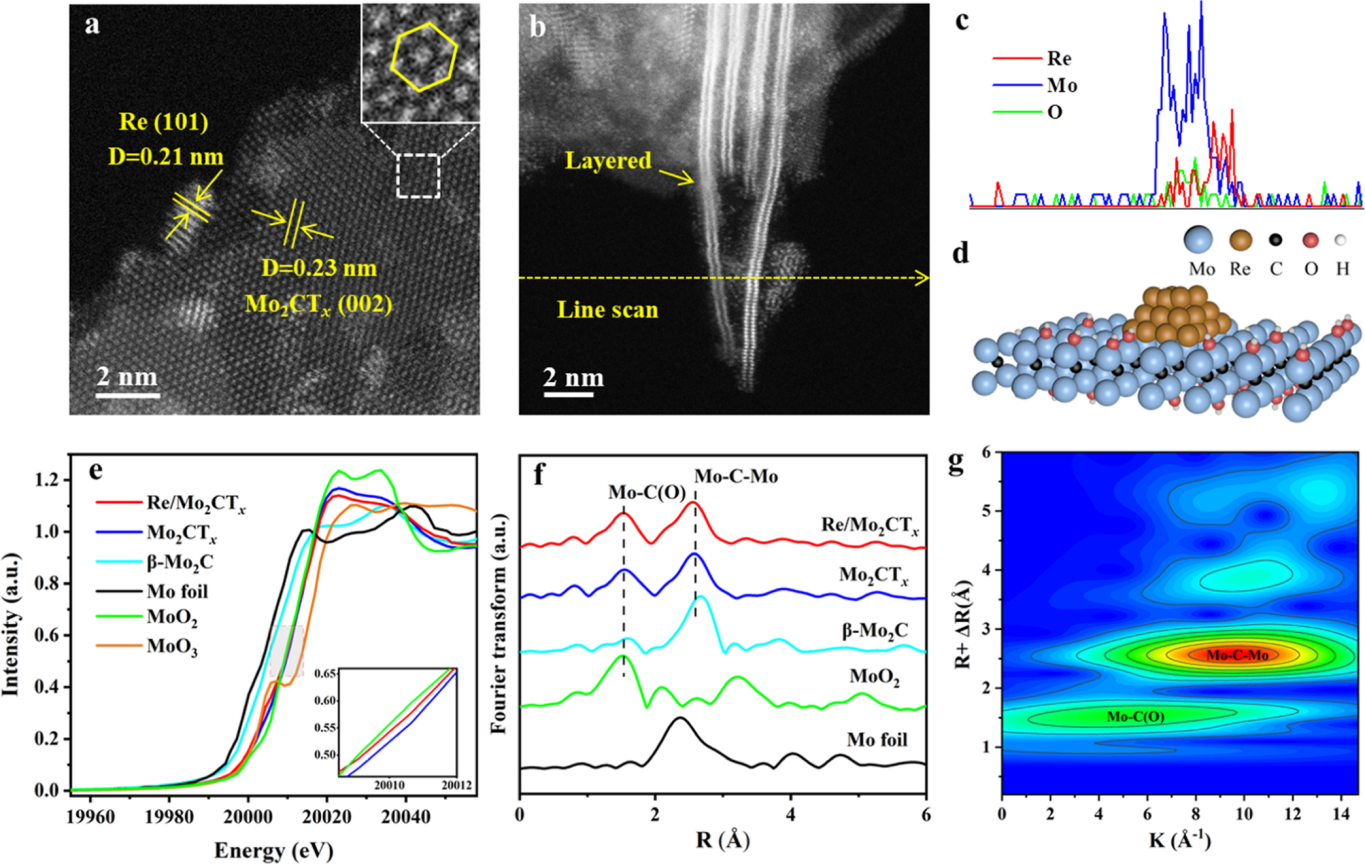
Geometric and electronic structures of Re/Mo_2_CT_*x*_. (a, b) Representative AC-HAADF-STEM images of Re/Mo_2_CT_*x*_. (c) Results of the line scan from (b). (d) Proposed model of Re/Mo_2_CT_*x*_. (e) Normalized Mo K-edge XANES and (f) *k*^2^ weighted Fourier transform EXAFS spectra in the *r*-space of Re/Mo_2_CT_*x*_ and reference samples. (g) Wavelet transformation for the *k*^2^-weighted EXAFS signal of the Re/Mo_2_CT_*x*_ catalyst.

A suite of elaborate characterization was employed to determine the structural properties of the best-performing Re/Mo_2_CT_*x*_ catalyst. High-resolution transmission electron microscopy (HRTEM) images (Figure S14a-c) show that the Re particles are highly dispersed throughout Mo_2_CT_*x*_. The statistical results indicate that the average particle size of the Re species is ∼2.3 nm (Figure S14d). The HRTEM shows that the average size of Re particles slightly increases from 2.3 to 2.5 nm after a long-term stability test (Figure S15), indicating a high stability of Re/Mo_2_CT_*x*_ for NH_3_ synthesis. Aberration-corrected high-angle annual dark-field scanning transmission electron microscopy (AC-HAADF-STEM) image (Figure 3a) clearly displays the lattice spacings of 0.21 and 0.23 nm, corresponding to the (101) and (002) crystal planes of Re metal and Mo_2_CT_*x*_,^37^ respectively. Moreover, the regular hexagonal crystal structure of Mo_2_CT_*x*_ can be observed over the magnified area (inset of Figure 3a), and the layered structure of Mo_2_CT_*x*_ can be clearly discerned (Figure 3b), which is consistent with the intrinsic characteristics of MXene.^38^ Furthermore, the line scan results (Figures 3b,c) demonstrate the loading of Re nanoparticles on the surface of Mo_2_CT_*x*_. Based on the 2D morphology of Mo_2_CT_*x*_ and the state of Re nanoparticles, a structural model of Re/Mo_2_CT_*x*_ was proposed, as depicted in Figure 3d.

To probe the fine structure of the Re and Mo species at the atomic level, a suite of *ex situ* synchrotron-based X-ray absorption spectroscopy experiments were performed. The spectra of Mo K-edge X-ray absorption near-edge structure (XANES) show that the absorption edge position of Mo_2_CT_*x*_ (Figure 3e) is close to that of MoO_2_, suggesting that the chemical state of Mo species in Mo_2_CT_*x*_ is near +4.^39,40^ After Re loading, a slight negative shift of the Mo absorption edge is observed over Re/Mo_2_CT_*x*_ (inset of Figure 3e). Meanwhile, the Re L-edge XANES spectra show that the charge state of Re is positive according to the Re foil and Re_2_O_7_ references (Figure S16). Meanwhile, X-ray photoelectron spectroscopy (XPS) results (Figure S17) show that the Mo 3d XPS spectra of Re/Mo_2_CT_*x*_ slightly shift a lower binding energy compared to Mo_2_CT_*x*_, which is attributed to electron donation from Re to Mo_2_CT_*x*_. Ultraviolet photoelectron spectroscopy (UPS) shows that the work function of Mo_2_CT_*x*_ is 5.7 eV (Figure S18), which is higher than that of metallic Re (4.3 eV), thus accounting for the electron transfer from Re to Mo_2_CT_*x*_.

The extended X-ray absorption fine structure (EXAFS) spectrum (Figure 3f) of Mo_2_CT_*x*_ presents two dominant peaks at 1.50 and 2.50 Å (not phase-corrected), corresponding to Mo–C(O) and Mo–C–Mo coordination, respectively.^41,42^ Notably, fitting results (Figure S19) show that the coordination numbers of Mo–C(O) over Mo_2_CT_*x*_ (6.6) and Re/Mo_2_CT_*x*_ (5.6) are significantly higher than that over β-Mo_2_C (3.0) (Table S2), which is attributed to the surface-terminated OH functional groups over MXene. The decrease of Mo–C(O) coordination number on Re/Mo_2_CT_*x*_ than Mo_2_CT_*x*_ implies that the loaded Re on Mo_2_CT_*x*_ can occupy the location of surface OH groups. Moreover, Mo–C(O) and Mo–C–Mo coordination of Re/Mo_2_CT_*x*_ was further demonstrated by wavelet transform (WT) analysis. The WT spectra over Re/Mo_2_CT_*x*_ exhibit lobes at 1.5 Å, 5.1 Å^–1^ and 2.6 Å, 9.5 Å^–1^, as displayed in Figure 3g, respectively, which are ascribable to Mo–C(O) and Mo–C–Mo compared with those of the β-Mo_2_C reference (Figure S20). In addition, the Re L-edge EXAFS spectra (Figure S21) show the presence of Re–Re (2.68 Å, not phase-corrected) and Re–O coordination (1.68 Å, not phase-corrected) over Re/Mo_2_CT_*x*_,^43^ and the slight positive shift of Re–Re coordination compared with that of Re foil reflects the interaction between Re and Mo_2_CT_*x*_.

### Roles of Re and Mo_2_CT_*x*_ Dual Sites in the Activation of N_2_ and H_2_

**Figure 4:**
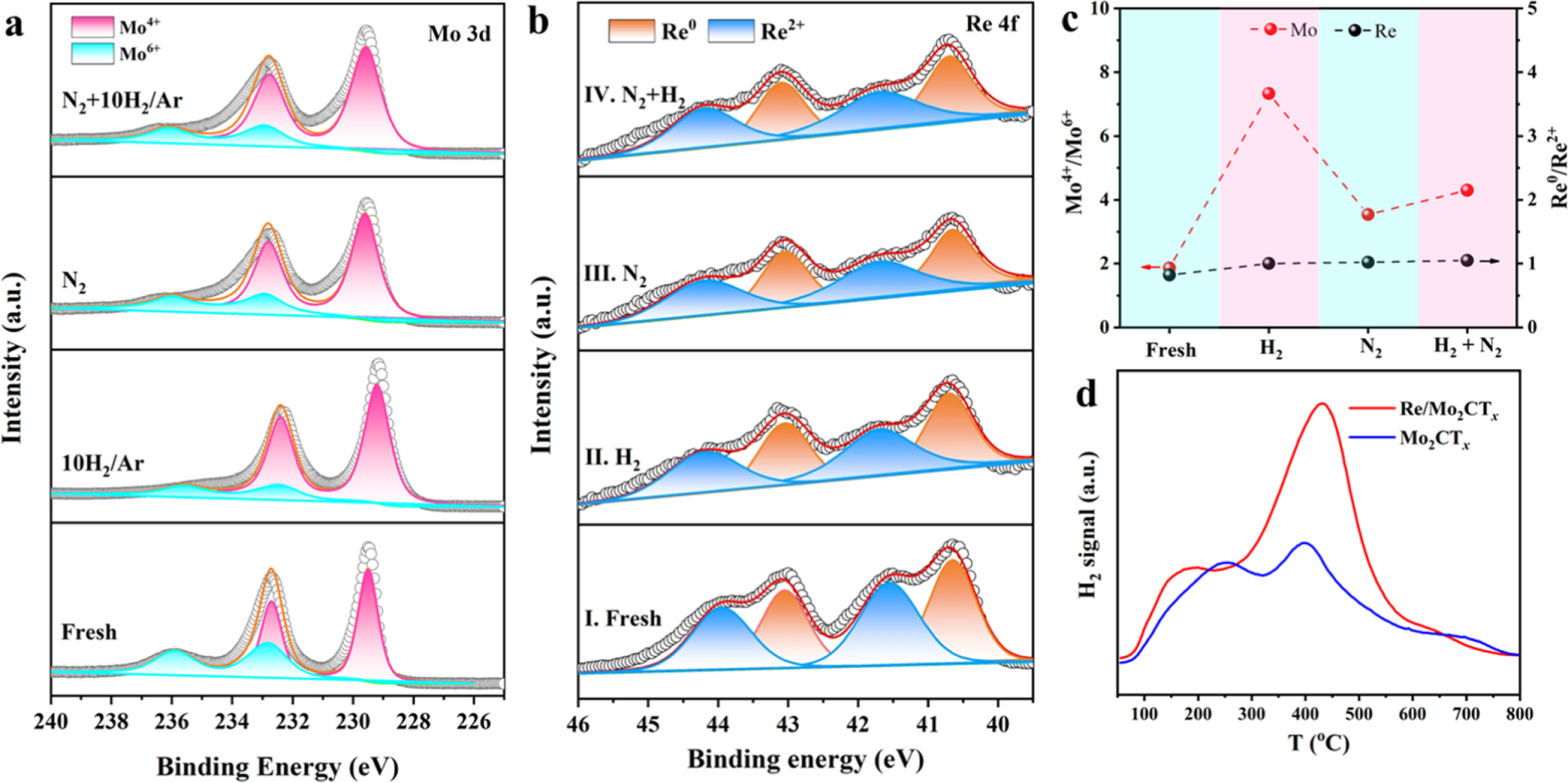
Dual sites of Re/Mo_2_CT_*x*_ for N_2_ and H_2_ activation. Quasi-*in situ* XPS spectra of (a) Mo 3d and (b) Re 4f over Re/Mo_2_CT_*x*_ under different gas environments at 400 °C for 2 h, (I) fresh sample, (II) 10%H_2_/Ar, (III) 2.5%N_2_/Ar, and (IV) 2.5%N_2_–7.5%H_2_/Ar. (c) Ratios of Mo^4+^/Mo^6+^ and Re^0^/Re^2+^ evolved from (a) and (b), respectively. (d) Signal of H_2_ desorption during the TPD experiment over Mo_2_CT_*x*_ and Re/Mo_2_CT_*x*_ after the coadsorption of N_2_ and H_2_ gases.

The roles of Re and Mo_2_CT_*x*_ in the activation of N_2_ and H_2_ for NH_3_ synthesis were investigated using quasi*-in situ* XPS experiments. Specifically, Re/Mo_2_CT_*x*_ was continuously treated under different atmospheres, and the evolution of the Mo 3d and Re 4f XPS spectra is displayed in Figure 4a,b, respectively. The Mo 3d spectra of fresh Re/Mo_2_CT_*x*_ can be deconvoluted into four characteristic peaks, where the pair at 229.5 and 232.7 eV is ascribed to Mo^4+^ and the pair at 232.8 and 236.0 eV belongs to Mo^6+^ species.^39,40^ According to the respective areas of the characteristic peaks, the proportions of Mo^4+^ and Mo^6+^ are 65% and 35%, respectively, giving a Mo^4+^/Mo^6+^ ratio of 1.9 (Figure 4c and Table S3). After pretreatment with H_2_ at 400 °C for 2 h, the Mo^4+^ content and the Mo^4+^/Mo^6+^ ratio increase to 88% and 7.3, respectively. Considering the H_2_-TPR results (Figure S3), it was deduced that the removal of surface oxygen species over Mo_2_CT_*x*_ by H_2_ reduction resulted in a higher exposure of Mo^4+^ sites. Upon further treatment in a N_2_ atmosphere at 400 °C for 2 h, the ratio of Mo^4+^/Mo^6+^ over Re/Mo_2_CT_*x*_ decreases to 3.5, indicating that Mo^4+^ can serve as active sites to activate N_2_ via Mo^4+^ + N_2_ → Mo^6+^ + 2N^–^, resulting in the formation of Mo^6+^-N species after electron transfer from Mo^4+^ to N_2_. Furthermore, the subsequent treatment of Re/Mo_2_CT_*x*_ in an N_2_–H_2_ atmosphere for 2 h would lead to an increase in the Mo^4+^/Mo^6+^ ratio, suggesting that a part of the Mo^6+^-N species undergoes hydrogenation to form NH_3_ and then restore Mo^4+^ sites. These results clearly demonstrate the dynamic transformation of the Mo^4+^ and Mo^6+^ sites for the conversion of N_2_ to NH_3_. It is noted that there is no substantial change in the number of Re^0^ and Re^2+^ species with the exposure to different atmospheres (Figure 4b,c), indicating that the Re species are not actively involved in N_2_ activation because N_2_ dissociation requires electron transfer from the active sites. This deduction is supported by the N_2_-TPD results. The N_2_-TPD-MS profile (Figure S22 and Table S4) shows almost no N_2_ desorption over pure Re metal, demonstrating the relatively weak effect of the Re species on N_2_ activation.

Furthermore, we investigated the cases in which Mo_2_CT_*x*_ and Re/Mo_2_CT_*x*_ were exposed to 25%N_2_–75%H_2_ feed gas, and the signals of H_2_ and N_2_ were monitored using an online MS. The desorption amount of H_2_ over Mo_2_CT_*x*_ was around 0.33 mmol g^–1^ (Figure 4d), which is around half that exposed to H_2_ atmosphere alone (Figure S4). The decreased amount of H_2_ desorption indicated that there was competitive adsorption between H_2_ and N_2_ in the co-fed case, and some of the sites for H_2_ activation were occupied by N_2_ molecules. We further verify the competitive adsorption of H_2_ and N_2_ over Mo_2_CT_*x*_ using density functional theory (DFT) calculations (a detailed description of the Mo_2_CT_*x*_ model is provided in Theoretical Calculations). Although H_2_ is easily dissociated over the surface of Mo_2_CT_*x*_,^44^ the sites where the adsorbed H atoms can be spontaneously occupied by N_2_ or N species (Figures S23 and S24), further demonstrate the preferential adsorption of N_2_ over Mo_2_CT_*x*_.

Note that the desorption amount of H_2_ over Re/Mo_2_CT_*x*_ (0.79 mmol g_Mo2CT*x*_^–1^) is obviously higher than that over Mo_2_CT_*x*_ (Figure 4d and Table S4), suggesting that the loading of Re could provide additional sites for H_2_ activation, which is consistent with previous reports that Re has a strong ability for H_2_ activation.^45,46^ Notably, the amount of H_2_ desorption over Re/Mo_2_CT_*x*_ is higher than the total amount over Mo_2_CT_*x*_ and Re metal separately (Figure S25), suggesting the possible occurrence of H-spillover over Re/Mo_2_CT_*x*_. Isothermal D_2_ isotopic exchange experiments show that the signals of H_2_ (*m*/*z* = 2) and HD (*m*/*z* = 3) simultaneously generate after the introduction of D_2_ over Re/Mo_2_CT_*x*_ (Figure S26), indicating that D_2_ can facilely dissociate into D* atoms and further react with the H or OH species of Mo_2_CT_*x*_ to form HD species. This demonstrates that the transfer and exchange of H/D species over Re/Mo_2_CT_*x*_ occur easily. To further demonstrate the existence of H-spillover over Re/Mo_2_CT_*x*_, a color change experiment was conducted on a mixture of WO_3_ and Re/Mo_2_CT_*x*_ because the blue H_*x*_WO_3_, which is called W-bronze, would be formed during the hydrogen reduction of yellow WO_3_. As depicted in Figure S27, hydrogen treatment of the mixture of WO_3_ and Re/Mo_2_CT_*x*_ led to a color change from light yellow to dark blue. In comparison, no distinct color change was observed for WO_3_. Similar phenomena are also observed over Ni/Mo_2_CT_*x*_ and Co/Mo_2_CT_*x*_ catalysts, certainly demonstrating the existence of H-spillover over NPMs/Mo_2_CT_*x*_. Taking into consideration the results of quasi-*in situ* XPS, it is deduced that the exposed Mo^4+^ sites of Mo_2_CT_*x*_ mainly serve as active sites for N_2_ adsorption and activation, while the Re entities play an essential role in promoting H_2_ activation, and H-spillover is favorable over Re/Mo_2_CT_*x*_.

### Insight into Structure–Activity Relationship over Re/Mo_2_CT_*x*_

**Figure 5:**
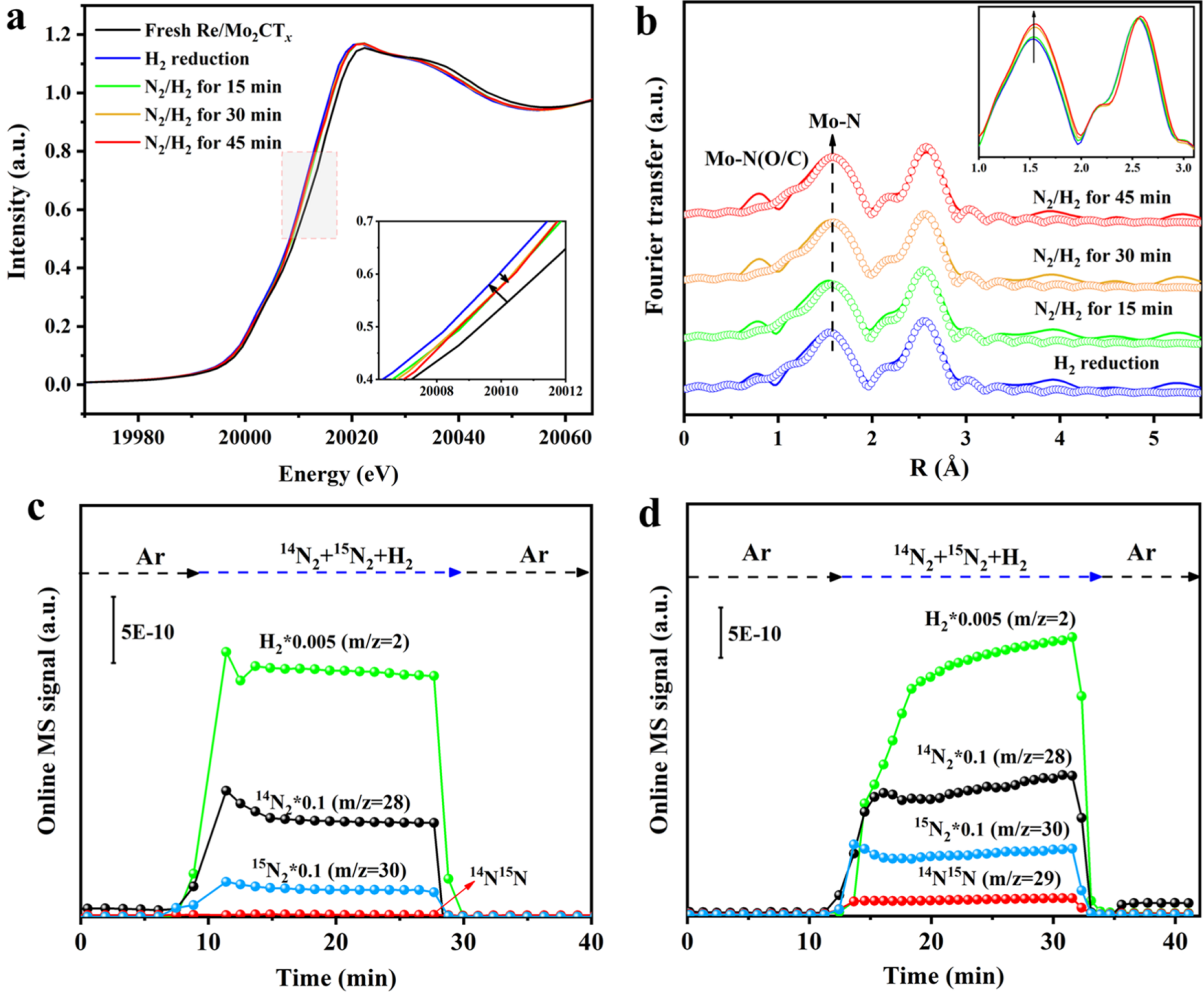
Structure–activity relationship over Re/Mo_2_CT_*x*_. (a) *In situ* normalized Mo K-edge XANES and (b) *k*^2^-weighted Fourier transform Mo K-edge EXAFS spectra in *r*-space and corresponding curve-fitting results of the Re/Mo_2_CT_*x*_ catalyst under 10%H_2_/Ar at 400 °C for 45 min and 2.5%N_2_–7.5%H_2_/Ar at 400 °C for different time-on-stream periods. Mass signals over (c) β-Mo_2_C and (d) Re/Mo_2_CT_*x*_ at 400 °C after the introduction of ^14^N_2_-^15^N_2_–H_2_, with a volume ratio of ^14^N_2_/^15^N_2_/H_2_ = 2:1:6.

The reaction process of NH_3_ synthesis over Re/Mo_2_CT_*x*_ was determined by cutting-edge operando techniques. Using a home-built cell (Figure S28), we performed *in situ* XANES and EXAFS measurements to further reveal the nature of the active sites during NH_3_ synthesis. As shown in Figure 5a, the *in situ* Mo K-edge XANES spectra of Re/Mo_2_CT_*x*_ show a negative shift in the absorption edge position after reduction by H_2_, indicating a decrease of the Mo valence state. This is in line with the results of quasi-*in situ* XPS, which demonstrated that the defunctionalization of Mo_2_CT_*x*_ by H_2_ resulted in higher exposure of Mo^4+^ active sites. As the reaction proceeded from 15 to 45 min under a N_2_–H_2_ mixture (Figure 5a) at 400 °C, the absorption edge position was positively shifted, suggesting an increase of the Mo valence state attributable to electron donation from Mo sites to the antibonding π* orbitals of N_2_. Moreover, the *in situ* Mo K-edge EXAFS spectra (Figure 5b and inset) clearly show that the first shell of the Mo–C(N) coordination number gradually increases from 5.6 to 6.3 with an increase of time-on-stream, while the second shell of Mo–C–Mo remain almost unchanged over Re/Mo_2_CT_*x*_ (Table S5). Considering the results of quasi-*in situ* XPS analysis, the increased coordination number in the first shell is attributed to the formation of Mo–N bonds after the activation of N_2_ over the Mo sites, further confirming that the Mo active sites of Mo_2_CT_*x*_ play a role in N_2_ activation in NH_3_ synthesis.

The dissociation of steady N≡N was usually regarded as RDS of NH_3_ synthesis over traditional Fe- or Ru-based catalysts.^6,33^ Given the strong ability of Mo_2_CT_*x*_ for N_2_ dissociation, we further investigate whether there is a change in RDS over Mo_2_CT_*x*_ and Re/Mo_2_CT_*x*_ by isotopic exchange experiments. After the introduction of the ^15^N_2_–^14^N_2_–H_2_ mixture, almost no ^14^N^15^N signal (*m*/*z* = 29) was detected over β-Mo_2_C (Figure 5c), while the ^14^N^15^N signal could be clearly discerned over Re/Mo_2_CT_*x*_ (Figure 5d) and Mo_2_CT_*x*_ (Figure S29). If the RDS is N–H formation, the nitrogen isotopic exchange reaction should readily proceed during the N–H_*x*_ (*x* = 1–3) bond formation.^47^ The appearance of the ^14^N^15^N signal clearly reveals the facile dissociation of ^14^N_2_ and ^15^N_2_ molecules to N* atoms over Mo_2_CT_*x*_, and then the accumulated ^14^N* and ^15^N* species recombine to generate ^14^N^15^N. These results not only demonstrate the dissociative route of N_2_ conversion to NH_3_ over Mo_2_CT_*x*_ and Re/Mo_2_CT_*x*_ but also indicate the shift of RDS from N_2_ dissociation over β-Mo_2_C to N–H_*x*_ formation over Mo_2_CT_*x*_ and Re/Mo_2_CT_*x*_.

Although the activation of N_2_ over Mo_2_CT_*x*_ is facile, the exceedingly strong adsorption of N species, as reflected by the high desorption temperature of N_2_ in N_2_-TPD (Figure 1d) would suppress the formation of N–H bonds and desorption of NH_3_ at low temperatures.^34,48^ To explore the synergistic effect of dual active sites on promoting N–H_*x*_ formation and NH_3_ desorption, Mo_2_CT_*x*_ and Re/Mo_2_CT_*x*_ were first exposed to an N_2_ atmosphere, and then an H_2_-temperature-programmed surface reaction (H_2_-TPSR) was performed to monitor the NH_3_ desorption signal. As shown in Figure S30, the initial and maximal desorption peaks of NH_2_ (*m*/*z* = 16) over Re/Mo_2_CT_*x*_ were distinctly shifted to lower temperatures compared with Mo_2_CT_*x*_, indicating the facile generation and desorption of NH_3_ after the addition of Re. These results are in accordance with the relatively more positive NH_3_ reaction order over Re/Mo_2_CT_*x*_ (−0.92) compared to that over Mo_2_CT_*x*_ (−1.63), indicating that there is a significantly decreased adsorption strength of NH_3_ over Re/Mo_2_CT_*x*_. In view of the role of Re for H_2_ activation and the presence of H-spillover behavior, which has been reported to associate with different sites and promote the hydrogenation of adsorbed species,^49,50^ it is reasonable to deduce that the H-spillover from Re to Mo_2_CT_*x*_ can promote the hydrogenation of adsorbed N species to form NH_3_. Meanwhile, the electron donation and H-spillover with the concurrent hydrogen proton–electron transfer^51^ from Re to Mo_2_CT_*x*_ can enrich the charge density on Mo_2_CT_*x*_ and induce a charge repulsion between Mo_2_CT_*x*_ and NH_3_ to accelerate NH_3_ desorption, thus resulting in the superior NH_3_ synthesis performance over Re/Mo_2_CT_*x*_.

### Theoretical Calculations on the Reaction Mechanism

**Figure 6:**
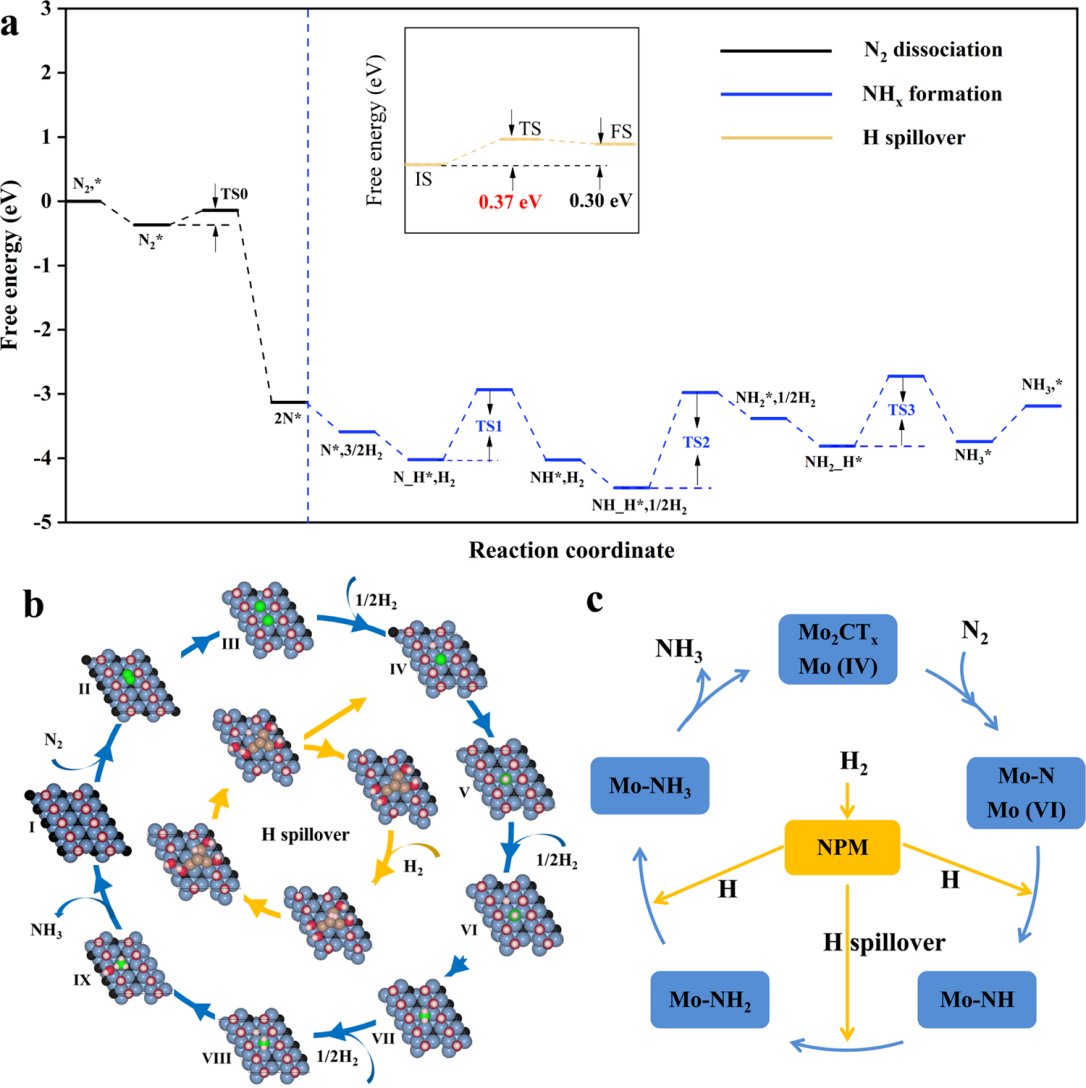
Theoretical calculations of the reaction mechanism. (a) Calculated free energy pathway of NH_3_ synthesis over Mo_2_CT_*x*_ and the H-spillover pathway from Re to Mo_2_CT_*x*_. (b) Structures of the key elementary steps (I–IX) for NH_3_ synthesis are shown in the outer circle and the structures of the H-spillover from Re to Mo_2_CT_*x*_ are shown in the inner circle. (c) Schematic of dual sites assisted by H-spillover over NPM/Mo_2_CT_*x*_ for NH_3_ synthesis. Color scheme: Mo atom: blue; Re atom: yellow; C atom: black; O atom: red; N atom: green; and H atom: white.

To gain insight into the reaction pathway of NH_3_ synthesis over the Re/Mo_2_CT_*x*_ catalyst, we performed DFT calculations. According to the XRD and Raman spectroscopy results, a slab model of Mo_2_COH terminated with 1/2 monolayer (ML) OH groups was constructed with the exposed (001) plane, as shown in Figure S31, which represents the removal of half of the OH groups from the surface. The model of Re-loaded Mo_2_COH (Re/Mo_2_COH) was proposed by loading a cluster of four Re atoms over Mo_2_COH, as shown in Figure S32. Then, the adsorption and activation behaviors of N_2_ over Mo_2_COH were first investigated. As shown in Figure S33, the calculation results indicate that the adsorption of N_2_ molecules on the Mo atoms of 2D Mo_2_COH is energetically favorable with an adsorption energy of −0.37 eV. The stable adsorption configuration of the double N atoms on the Mo_2_COH surface was also examined, and the calculated adsorption energies ranged from −1.98 to −3.13 eV (Figure S34), which are less negative than those of the reported LaCoSi electride catalyst (−3.45 eV).^11^ These results indicate that Mo_2_COH can be promising for N_2_ activation because a suitable N-surface interaction can avoid nitrogen poisoning. The transition state from a lateral N_2_-adsorbed configuration (Figure S33b) to the most stable double N configuration (Figure S34b) was determined by performing nudged elastic band (NEB) calculations. As shown in Figure 6a, the dissociation barrier of N≡N was calculated to be only 0.23 eV over Mo_2_CT_*x*_, indicating facile activation of N_2_ over Mo_2_COH. Bader charge analysis shows that a single N atom will get 0.70 and 1.14 electrons mainly from Mo atoms when N_2_ and 2N adsorb on the surface of Mo_2_CT_*x*_, respectively (Figure S35). The above results are in good agreement with the results of the *in situ* EXAFS (Figure 5b), further demonstrating the role of Mo sites in N_2_ activation. More importantly, the calculated very low activation energy of N_2_ suggests that N_2_ activation may not be the limiting step in NH_3_ synthesis.

Our experimental measurements revealed that the loading of Re clusters on the surfaces of Mo_2_COH can dramatically improve the catalytic performance, which suggests that Re loading can speed up the limiting step. Further calculations showed that H_2_ molecules can spontaneously dissociate on both the Re cluster and Mo_2_COH. However, the adsorption of hydrogen on the Re cluster is stronger than that on Mo_2_COH. The calculated adsorption energies of the H atom on Re/Mo_2_COH and Mo_2_COH are −1.31 and −1.01 eV/H, respectively. Therefore, it can be hypothesized that Re clusters supported on Mo_2_COH can accelerate NH_3_ catalytic performance by increasing the supply of H atoms for the NH_3_ formation reaction. To verify this hypothesis, we studied the H-spillover behavior over Re/Mo_2_CT_*x*_. It was found that the spillover of H from the Re cluster to Mo_2_CT_*x*_ only needs an energy barrier of 0.37 eV, as illustrated in Figure 6a and Table S6, implying the easy migration of activated H species to the Mo_2_CT_*x*_ surface. Furthermore, a first-principles molecular dynamics simulation was carried out to show the adsorption and dissociation of H_2_ on the Re cluster. The initial configuration was constructed by placing an H_2_ molecule about 2 Å above the Re cluster (Figure S36). One can see that the dissociation quickly occurred because the distance between H–H atoms increased dramatically. One H atom was first adsorbed on the Re cluster and moved quickly to the Mo_2_CT_*x*_ surface, demonstrating that hydrogen spillover is relatively facile on Re/Mo_2_CT_*x*_. Therefore, it was concluded that the facile H* spillover guarantees an adequate H source that is conducive to N–H and NH_3_ formation over the Mo_2_CT_*x*_ surface.

Furthermore, the subsequent hydrogenation of the dissociated N species to form NH_*x*_ (*x* = 1–3) was studied. It is worth noting that a slab model with one adsorbed N atom was used for the NH_*x*_ formation reaction for ease of calculation. As displayed in Figure 6b (IV–V) and Table S7, the first hydrogenation step for the generation of NH* must overcome an energy barrier of 1.09 eV, which is higher than that of N_2_ dissociation. The calculated energy barriers for further hydrogenation to form NH_2_* and NH_3_* are 1.48 and 1.09 eV, as shown in Figure 6b (VI-IX), respectively. Detailed information regarding the calculated routes of NH_*x*_ formation is shown in Figures S37–39. These results demonstrate that the dissociation of N_2_ is easier than that of the N–H bond formation, and the RDS of NH_3_ synthesis is the formation of NH_2_* species rather than the direct dissociation of the N≡N bond. This conclusion is consistent with the results of the isotopic exchange experiments, as shown in Figure 5d. DFT calculations further demonstrate that the synergistic effect of Re and Mo_2_CT_*x*_ dual sites with the assistance of H-spillover enables facile NH_3_ synthesis over Re/Mo_2_CT_*x*_ under mild conditions.

## Discussion

The development of advanced catalysts with high efficiency under mild conditions is a long-term pursuit for NH_3_ synthesis, especially for the potential application of the *e*HB process.^7^ To date, despite being promising substitutes for noble metal Ru catalysts, NPM catalysts suffer from low NH_3_ synthesis performance due to the inverse relationship of the dissociation barrier of N_2_ and the desorption energy of NH_*x*_ on NPMs. In the present study, we mediated Mo_2_CT_*x*_ with NPM, which combines the positive effects of dual sites and H-spillover to address the major obstacle and achieve a performance comparable to that of Ru-based catalysts. Our studies show that the surface-terminated oxygen group (T_*x*_) of Mo_2_CT_*x*_ can be partially reduced to expose a highly active 2D platform for N_2_ activation (Figure 6c). Nonetheless, the exposed Mo ions act as the active sites for N_2_ while suppressing H_2_ activation and N–H_*x*_ formation. With the assistance of Re, H_2_ can be easily activated and the further H-spillover from Re to Mo_2_CT_*x*_ can facilitate the hydrogenation of N adatoms to form N–H_*x*_ species. Meanwhile, electron transfer from Re to Mo_2_CT_*x*_ can enrich its negative charge density, inducing charge repulsion with NH_3_ to accelerate its desorption (Figure 3e and Figure S30). Thus, the easy activation of N_2_ and the facile desorption of NH_3_ species were simultaneously realized via the strategy of dual active sites bridged by H-spillover; that is, the bottleneck of NH_3_ synthesis was decoupled over Re/Mo_2_CT_*x*_. Similarly, Ni and Co metals are known to possess a strong ability for H_2_ activation and can act similarly to the Re metal in this aspect,^15^ but they are relatively inert for N_2_ activation, as demonstrated by N_2_/H_2_-TPD experiments (Figure S40). With the synergistic effect of Mo_2_CT_*x*_, there is also superior NH_3_ synthesis performance over Ni/Mo_2_CT_*x*_ and Co/Mo_2_CT_*x*_ under mild conditions. It is worth noting that the strategy of combining dual sites with H-spillover significantly differs from that of Co_3_Mo_3_N and Ni/LaN nitride systems that rely on nitrogen vacancies for N_2_ activation.^14,15^ Meanwhile, the assistance of H-spillover can break the spatial limitation of dual active sites that need close connection to ensure the generation of products, thus increasing the available and effective dual active sites for NH_3_ formation. It is envisaged that the findings of the present investigation will enable more NPMs to be utilized as potential catalyst components for efficient NH_3_ synthesis under mild conditions.

## Conclusions

In summary, we developed NPMs (Co, Ni, and Re)-loaded Mo_2_CT_*x*_ MXenes as efficient catalysts for NH_3_ synthesis under mild conditions. Our studies demonstrated that Mo^4+^ sites exposed by the partial detachment of the surface-terminating group (T_*x*_) over Mo_2_CT_*x*_ work as the key active sites for N_2_ dissociation, while NPMs are responsible for the activation of H_2_. Meanwhile, the RDS of NH_3_ synthesis over these NPM/Mo_2_CT_*x*_ catalysts is not the dissociation of N_2_ but rather the formation of NH_2_*. The H-spillover and electron transfer from NPMs to Re to Mo_2_CT_*x*_ can promote the hydrogenation of dissociated N species and desorption of NH_3_, thus enabling NH_3_ synthesis with high efficiency. The strategy of dual sites bridged by H-spillover can circumvent the bottleneck of NH_3_ synthesis and shed light on the design of advanced NPM-based catalysts for NH_3_ synthesis under mild conditions.

## Experimental Section

### Chemicals and Materials

Mo_2_C (≥99.95%) and Mo metal (≥99.5%) were purchased from Macklin Co., Ltd. Mo_2_Ga_2_C (≥99%) was purchased from Beike 2D Materials Co., Ltd. MoO_2_ (≥99%), MoO_3_ (≥99.5%), NH_4_ReO_4_ (≥99%), Co(NO_3_)_2_·6H_2_O (≥99.99%), and Ni(NO_3_)_2_·6H_2_O (≥99%) were from Aladdin Biochemical Technology. Notably, Re was cheaper by an order of magnitude than Ru, irrespective of its lower abundance. D_2_ and 5% ^15^N_2_/Ar gas were obtained from Cambridge Isotope Laboratories.

### Catalyst Preparation

Mo_2_CT_*x*_ MXene was synthesized via the etching of a Mo_2_Ga_2_C precursor using a hydrofluoric acid (HF) solution. In detail, 2 g of Mo_2_Ga_2_C was added to 60 mL of 40% HF under continuous magnetic stirring at 65 °C for 72 h. Then, the product was washed with deionized H_2_O and centrifuged at 4000 rpm several times until the pH was >6. Subsequently, the clay-like sediment was dispersed into 1 L of alkaline solution containing 0.2 mol L^–1^ NaOH and 1 mol L^–1^ NH_3_·H_2_O, and the resulting mixture was subjected to sonication for 1 h to remove the F anions of MXene. Afterward, the suspension was filtered and washed with deionized water. Finally, the sediment was vacuum freeze-dried to obtain Mo_2_CT_*x*_ MXene with a layered structure.

NPMs/Mo_2_CT_*x*_ (NPMs = Re, Ni, and Co) catalysts were synthesized using the impregnation method. For Re/Mo_2_CT_*x*_, a quantity of NH_4_ReO_4_ was dissolved in 5 mL of deionized water. Then, NH_4_ReO_4_ solution was added dropwise to 1 g of Mo_2_CT_*x*_, and the resulting mixture was dried using an infrared lamp. After that, Re/Mo_2_CT_*x*_ was obtained by heating the mixture at a rate of 2 °C min^–1^ to 500 °C for 2 h under 10 vol % H_2_/Ar. Notably, to avoid over-oxidation of the sample by air, the catalyst was extracted after a passivating treatment with 1 vol %O_2_/Ar for 6 h at room temperature. Therefore, the Re in Re/Mo_2_CT_*x*_ was controlled to 5, 10, and 15 wt %, respectively. Co/Mo_2_CT_*x*_ and Ni/Mo_2_CT_*x*_ catalysts were prepared using the same procedure, except that Co(NO_3_)_2_·6H_2_O and Ni(NO_3_)_2_·6H_2_O were used as precursors.

The Mo_2_N reference was synthesized via the nitridation of MoO_3_ using NH_3_ gas, as described elsewhere.^52^ The Co_3_Mo_3_N reference was synthesized according to the method described by Kojima and Aika.^53^ Cs–Ru/MgO was prepared by impregnation methods, where the Cs and Ru contents were 5 and 5 wt %, respectively.

### Evaluation of Catalytic Performance

The catalytic activities for NH_3_ synthesis over the as-prepared catalysts were measured in a pressurized fixed-bed reactor. The details of the activity measurements, reaction orders of N_2_, H_2_, and NH_3_, and methanation experiments are shown in Supporting Information.

### Catalyst Characterization

The details of the X-ray diffraction (XRD), Raman analysis, inductively coupled plasma-atomic emission spectroscopy (ICP-AES), X-ray photoelectron spectroscopy (XPS), scanning electron microscopy (SEM), high-resolution transmission electron microscopy (HRTEM), high-angle annular dark-field scanning transmission electron microscopy (HAADF-STEM), energy-dispersive X-ray spectroscopy (EDX), and aberration-corrected high-angle annular dark-field scanning transmission electron microscopy (AC-HAADF-STEM) are shown in Supporting Information. Notably, before measurement of HRTEM, HAADF-STEM, and AC-HAADF-STEM, the catalyst was treated with 10%H_2_/Ar at 400 °C for 2 h.

### Quasi-*In Situ* XPS Measurements

Quasi-*in situ* XPS experiments were conducted using a Thermo Scientific Escalab 250Xi instrument. Specifically, the sample was subsequently treated with different gases for several hours before each measurement. Initially, Re/Mo_2_CT_*x*_ was reduced with 10%H_2_/Ar at 400 °C for 2 h, and then the sample was transferred to the analysis chamber at room temperature for XPS measurements. Afterward, the sample was treated stepwise with 2.5%N_2_/Ar and 2.5%N_2_–7.5%H_2_/Ar at 400 °C for 2 h. The spectra of the catalysts were recorded after each treatment. The binding energies were referenced to the C 1s peak of adventitious carbon set at 284.8 eV.

### *Ex Situ* XANES and EXAFS

The X-ray absorption spectra (XAS), including XANES and EXAFS at the Mo K-edge and Re L-edge of the samples, were measured at beamline 1W1B of the Beijing Synchrotron Radiation Facility in China. The output beam was selected using a Si(311) monochromator for Mo K-edge absorption spectra and a Si(111) monochromator for Re L-edge absorption spectra, and the energy was calibrated against Mo and Re foils. The data for the as-prepared catalysts and reference materials were collected at room temperature in the transmission mode.

### *In Situ* XANES and EXAFS

*In situ* XANES and EXAFS analyses were performed at the 1W1B beamline of the Beijing Synchrotron Radiation Facility. The apparatus for the experiment was provided by the Beijing Institute of Chemical Industry of Sinopec and contained an *in situ* cell and system for gas circulation and sample heating. The catalyst sample was pressed into uniform flakes to allow the transmission of signals to pass from the sample to the detector. The *in situ* XANES and EXAFS data were collected at 400 °C in the presence of 10%H_2_/He or N_2_–H_2_ (V_N2_/V_H2_ = 1:3) as a function of time.

### D_2_ Isotopic Exchange Experiment

The D_2_ isotopic exchange experiments were conducted using an Autochem 2920 instrument equipped with an online mass spectrometer. The sample was then treated with 10%H_2_/Ar at 500 °C for 2 h. Then, the temperature was lowered to 400 °C, and the sample was exposed to 5%D_2_/Ar. The H_2_ (*m*/*z* = 2), HD (*m*/*z* = 3), and D_2_ (*m*/*z* = 4) signals were recorded as a function of time.

### ^14^N_2_, ^15^N_2_, and H_2_ Isothermal Isotopic Labeling Experiments

Isothermal isotopic labeling experiments were performed using an Autochem 2920 instrument equipped with an online mass spectrometer. The sample was treated with 10%H_2_/Ar at 500 °C for 2 h. Then, the temperature was lowered to 400 °C, and the sample was exposed to ^14^N_2_-^15^N_2_–H_2_ with a volume ratio of ^14^N_2_/^15^N_2_/H_2_ = 2:1:6. Meanwhile, the H_2_ (*m*/*z* = 2), ^14^N_2_ (*m*/*z* = 28), ^14^N^15^N (*m*/*z* = 29) and ^15^N_2_ (*m*/*z* = 30) signals were recorded as a function of time.

### DFT Calculations

First-principles calculations were performed using density functional theory (DFT) methods, as implemented in the Vienna Ab-initio Simulation Package (VASP).^54^ The generalized gradient approximation (GGA) with the PBE exchange-correlation functional was used.^55^ The projector augmented wave (PAW) method was used to describe the ionic cores.^56^

A 4 × 4 × 1 Mo_2_COH slab model with an exposed (001) plane was built with a vacuum layer larger than 35 Å. The Re/Mo_2_COH model was constructed using a tetrahedral cluster of 4 Re atoms loaded on the Mo_2_COH model. For the structural optimization of the Mo_2_COH and Re/Mo_2_COH models, Brillouin zone sampling was used with a grid spacing density of 2π × 0.03 Å^–1^.The plane-wave cutoff energy, convergence criterion of self-consistent iterations, and force on each atom were set to 500 eV, 1 × 10^–5^ eV, and −0.03 eV/Å, respectively. All of the atoms were fully relaxed during the optimization calculations.

To study the entire formation path of NH_3_, the X/slab structures (X stands for H, N, N_2_, NH_*x*_ (*x* = 1–3), and NH_*y*__H (*y* = 0,1,2), and slab stands for the Mo_2_COH and Re/Mo_2_COH model) were optimized using the same parameters as those for the Mo_2_COH structural optimization mentioned above. The adsorption energies of the X species (*E*_ad_(X)) on the Mo_2_COH and Re/Mo_2_COH models were calculated using the following equation
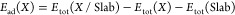
where *E*_tot_(X/Slab) is the total energy of the slab model with X adsorption, and *E*_tot_(X) and *E*_tot_(Slab) are the total energies of the X species and slab model, respectively. The half energy of N_2_ and H_2_ molecules were used as the energies of nitrogen and hydrogen atoms, respectively.

The climbing image nudged elastic band (CI-NEB)^57^ and dimer^58^ methods were employed to search for the transition states (TS) of the elementary steps of ammonia synthesis. Brillouin zone sampling used a grid with a spacing of 2π × 0.03 Å^–1^. The cutoff energy for the TS calculations was set to 450 eV, with an energy precision of 1 × 10^–5^ eV. All of the atoms were allowed to move during the TS calculations until the force on each atom reached −0.05 eV/Å. The predicted transition states were further confirmed using vibrational frequency analysis.

First-principles molecular dynamics (FPMD) calculations were performed using VASP to simulate the process of H_2_ spontaneous dissociation in Re/Mo_2_COH. An NVE ensemble with a time step of 3.0 fs was used for FPMD simulations.

## Supporting Information Available

Catalyst characterization and reaction activity (Figures S1–S40 and Tables S1–S7) (PDF)
