# Supplementary material for: Precious-Metal-Free Mo-MXene Catalyst Enabling Facile Ammonia Synthesis Via Dual Sites Bridged by H-Spillover
Source: J Am Chem Soc. 2024 Aug 12;146(33):23054–66. doi: 10.1021/jacs.4c03998 (PMC11345764; doi:10.1021/jacs.4c03998)
Supplement: Supplementary file 1 — ja4c03998_si_001.pdf [file ja4c03998_si_001.pdf]

Supporting Information

Precious-Metal-Free Mo-MXene Catalyst Enabling Facile Ammonia Synthesis via Dual Sites Bridged by H-Spillover

Yanliang Zhou[1#], Lili Liang[2#], Congying Wang[1], Fuxiang Sun[1], Lirong Zheng[3], Haifeng Qi[4], Bin Wang[5], Xiuyun Wang[1]*, Chak-tong Au[1], Junjie Wang[2]*, Lilong Jiang[1]* and Hideo Hosono[6]*

[1]National Engineering Research Center of Chemical Fertilizer Catalyst, Fuzhou University, Fuzhou, 350002, China.

[2]State Key Laboratory of Solidification Processing, School of Materials Science and Engineering, Northwestern Polytechnical University, Xi'an, 710072, China.

[3]Institute of High Energy Physics, Chinese Academy of Sciences, Beijing 100049, China.

[4]Leibniz-Institut für Katalyse e.V., Rostock, 18059, Germany.

[5]Sinopec Beijing Research Institute of Chemical Industry, Beijing, 100013, China.

[6]MDX Research Center for Element Strategy, Tokyo Institute of Technology, Yokohama, Kanagawa 226-8503, Japan.

[#]These authors contributed equally to this work.

*Email: xywangfzu@163.com (X. W.); wang.junjie@nwpu.edu.cn (J. W.); jll@fzu.edu.cn (L. J.); hosono@mces.titech.ac.jp (H. H.)

1. Experimental section

1.1 Evaluation of Catalytic Performance. In detail, 0.2 g of a catalyst (20−40 mesh) was diluted with quartz powder and loaded into the stainless steel reactor tube (i.d.= 10 mm). Before measurement, the catalysts were treated in a flow of 25%N_2-75%H_2 at 500 °C for 2 h. Then, the catalyst performance was evaluated at a WHSV of 60 000 mL g^{-1} h^{-1} and at a given pressure and temperature. The outlet NH_3 concentration was measured using a known amount of diluted H_2SO_4 solution (0.2 mol L^{-1}), as well as being analyzed by ion chromatography (Thermo Scientific, DIONEX, ICS-600). Apparent activation energies of the as-prepared catalysts were calculated from Arrhenius plots of the NH_3 synthesis rates in the temperature range of 300−400 °C.

The NH_3 synthesis rate (R) was calculated as follows:

$$R = \frac{\mathrm{X} \cdot V_L}{m_{cat} \cdot M_{NH3} \cdot \mathrm{t}} \qquad (1)$$

Where x (ppm) represents the content of NH_3 measured by ion chromatography; V_L (L) represents the volume of diluted H_2SO_4 solution; m_{cat} (g) represents the mass of catalyst; M_{NH3} (g mol^{-1}) represents the molecular weight of NH_3; t represents the absorption time of outlet gases in diluted H_2SO_4 solution.

Limited by the thermodynamics of NH_3 synthesis, at 400 °C and 1 MPa with a flow of 25%N_2-75%H_2, the equilibrium concentration of NH_3 synthesis is only 4.1%. Due to the low NH_3 yiled (≤5%), the volume change in NH_3 synthesis process is negligible. The outlet concentration of NH_3 (C_{NH3}) and the conversion of N_2 (Con_{N2}) can be calculated as follows:

$$C_{NH3} = \frac{\mathrm{R} \cdot V_m}{\mathrm{WHSV}} \qquad (2)$$

$$Con_{N2} \approx 2C_{NH3} \qquad (3)$$

Where R (mol g^{-1} h^{-1}) represents the NH_3 synthesis rate; V_m (24.8 L mol^{-1} at 25 °C) represents the molar volume of a gas; WHSV (L g_{cat}^{-1} h^{-1}) represents the weight hourly space velocity of reactants.

The reaction orders of N_2, H_2, and NH_3 were determined by the equation:

$$r = k\, P_{N2}{}^{\alpha}\, P_{H2}{}^{\beta}\, P_{NH3}{}^{\gamma} \qquad (4)$$

Where r is the reaction rate and k the reaction constant. P_{N2}, P_{H2}, and P_{NH3} is the partial pressures of N_2, H_2, and NH_3; and α, β, and γ *is* the reaction order of N_2, H_2, and NH_3, respectively.

To determine NH_3 reaction order (γ), P_{N2} and P_{H2} are fixed, and the flow rate of feed gas was varied as 50, 100, 150, 200, 250 and 300 mL min^{-1}. The data were analyzed according to Aika's method[1]. To measure reaction orders for N_2 and H_2, P_{H2} or P_{N2} is fixed, and Ar gas was taken as the diluent to assure a total flow of 200 mL min^{-1}. The constituent gases of the reactant (N_2, H_2, Ar) in volume fraction were as follows: (12.5%, 35%, 52.5%), (25%, 35%, 40%), (35%, 35%, 30%), (45%, 35%, 20%), (55%, 35%, 10%) and (62.5%, 35%, 2.5%) for the measure of N_2 order, and (25%, 50%, 12.5%), (25%, 37.5%, 25%), (25%, 25%, 37.5%) and (25%, 12.5%, 62.5%) for H_2 order. The effect of NH_3 concentration on the reaction order of N_2 or H_2 was eliminated by plotting $\log(r) - \gamma\log(P_{NH3})$ versus $\log(P_{N2})$ or by plotting $\log(r) - \gamma\log(P_{NH3})$ versus $\log(P_{H2})$ as reported by Iriawan, et al[2].

For the determination of the possibility of methanation, 0.2 g of Re/Mo_2CT_x was exposed to a flow of 25% N_2-75% H_2 at 400 °C and 1 MPa. The outlet CH_4 concentration was detected using an online GC-mass spectrometer (GCMS-QP2010 SE).

1.2 Catalyst Characterization. X-ray diffraction (XRD) patterns were collected on a PANalytical X'Pert Pro diffractometer using Cu K_α radiation source. Raman analysis was performed under ambient conditions on a Renishaw spectrometer employing a laser beam of λ = 532 nm. X-ray photoelectron spectroscopy (XPS) was carried out on Thermo Scientific

Escalab 250Xi equipped with a monochromatic Al Kα source (hν = 1486.6 eV) as the X-ray source. Thermogravimetric analysis (TGA) was conducted on a simultaneous thermal analyzer (Setsys Evolution) under Ar atmosphere from 50−800 °C.

Scanning electron microscopy (SEM) was performed on a Hitachi Model S-4800 instrument. Transmission electron microscopy (TEM), high resolution TEM (HRTEM), high-angle annular dark-filed scanning transmission electron microscopy (HAADF-STEM) and energy-dispersive X-ray spectroscopy (EDX) were conducted on a JEM-2010 instrument. Aberration-corrected high-angle annual dark-filed scanning transmission electron microscopy (AC-HAADF-STEM) analysis was performed on a JEOL JEM-ARM 200 F instrument equipped with a CEOS probe corrector.

2. Supporting Figures

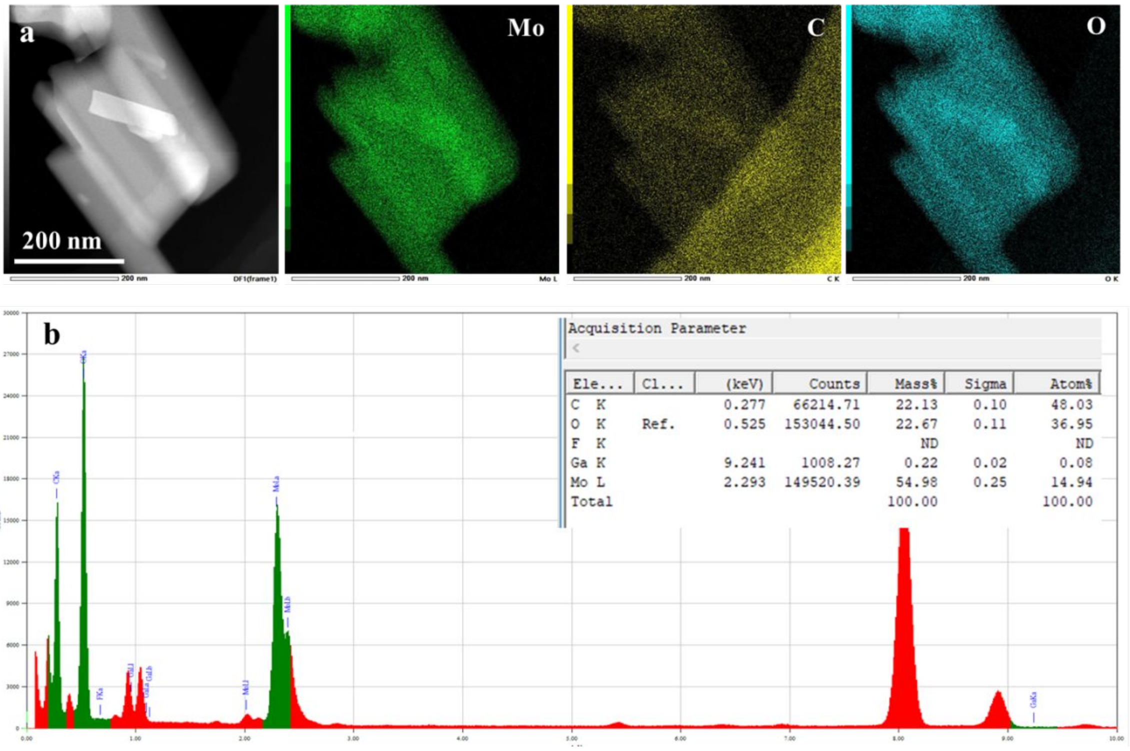

Figure S1. (a) HAADF-STEM and EDX images of Mo_2CT_x, (b) element spectra and mass content of Mo, C, O, Ga, and F over Mo_2CT_x.

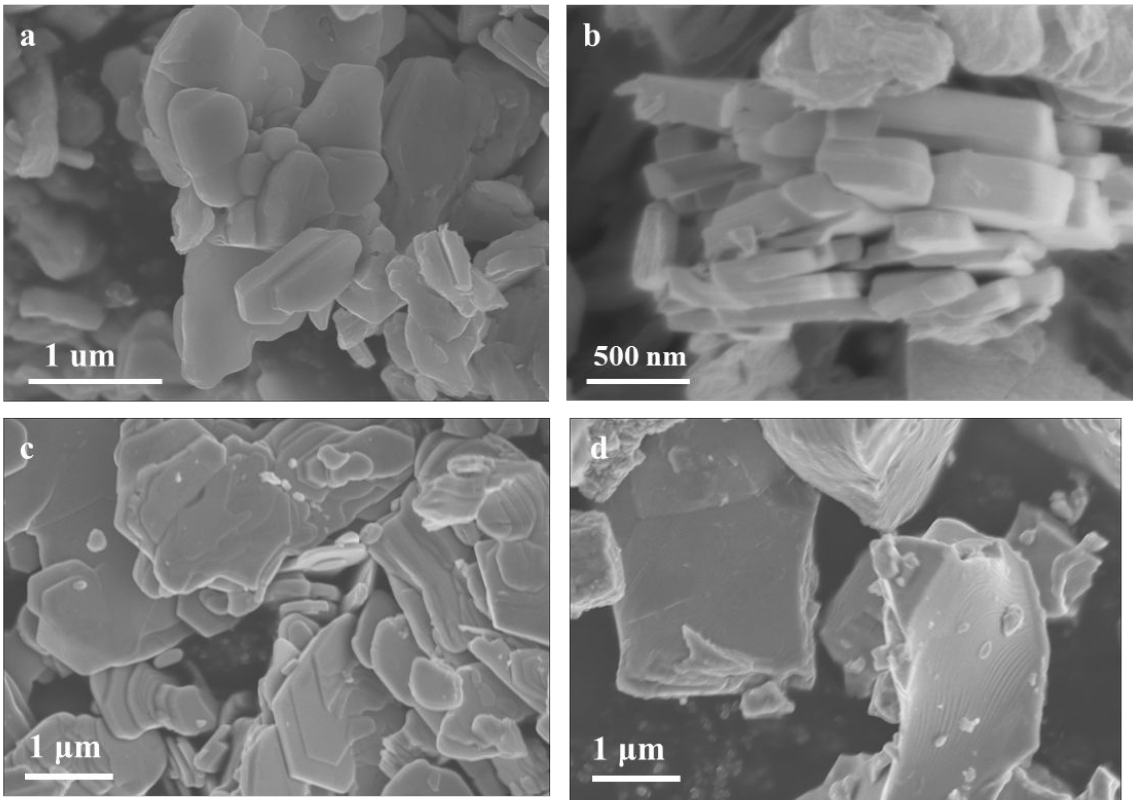

Figure S2. SEM images of (a-b) Mo_2CT_x, (c) Mo_2Ga_2C, and (d) β-Mo_2C.

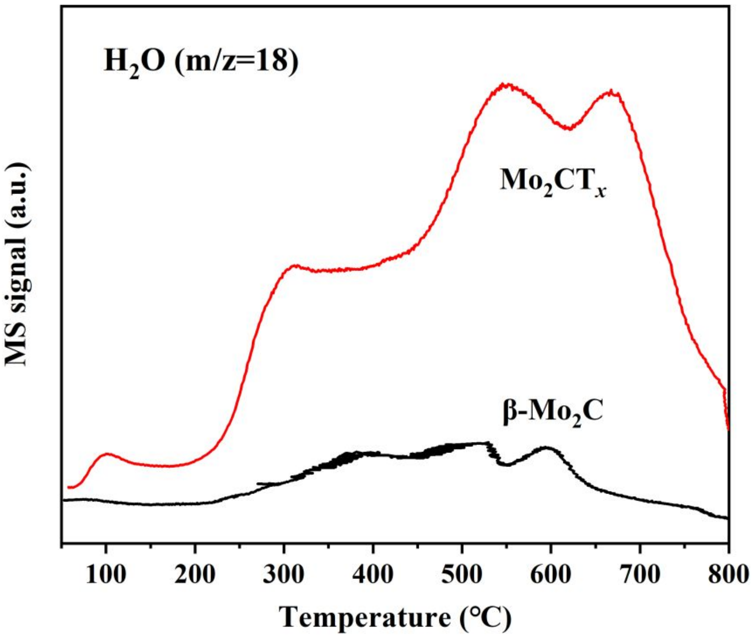

Figure S3. H_2-TPR over Mo_2CT_x and β-Mo_2C with MS monitoring the water signal.

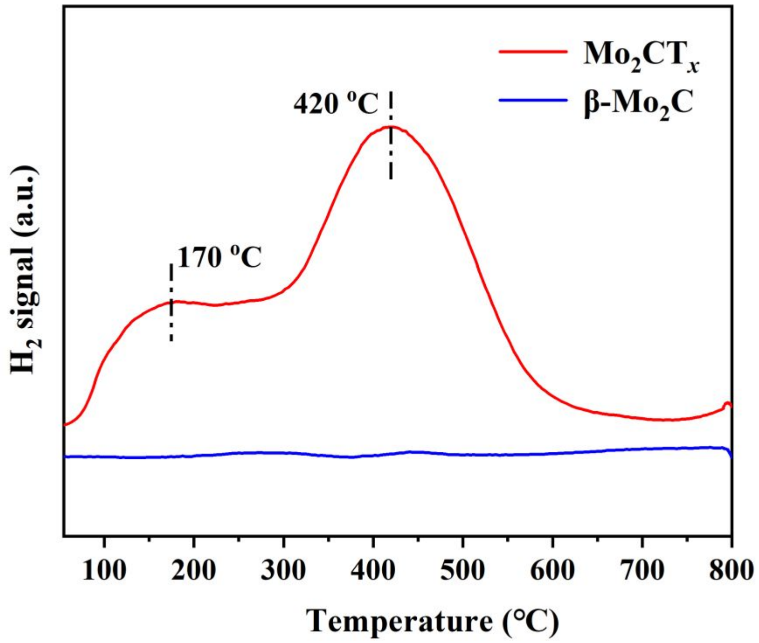

Figure S4. H_2-TPD profiles of Mo_2CT_x and β-Mo_2C recorded by on-line MS detection.

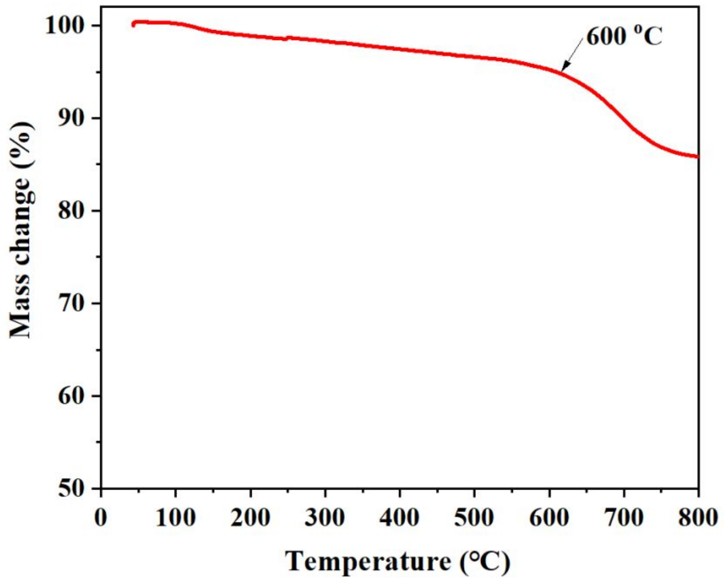

Figure S5. Thermogravimetric analysis (TGA) of the fresh Mo_2CT_x under Ar atmosphere.

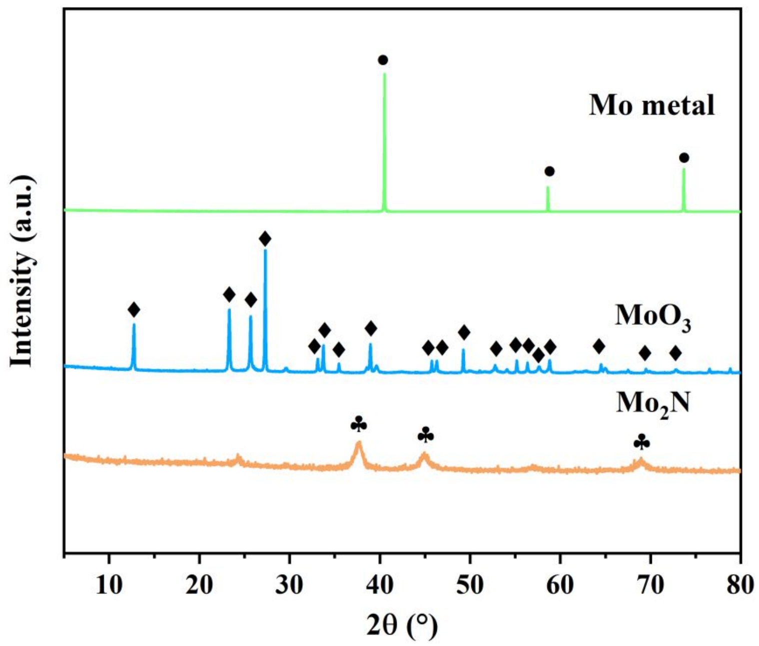

Figure S6. XRD patterns of Mo metal, MoO_3, and Mo_2N.

The JCPDS cards of Mo, MoO_3, and Mo_2N are no. 00-042-1120, no. 00-005-0508, and no. 00-025-1366, respectively. Over these samples, no obvious impurity phases were observed.

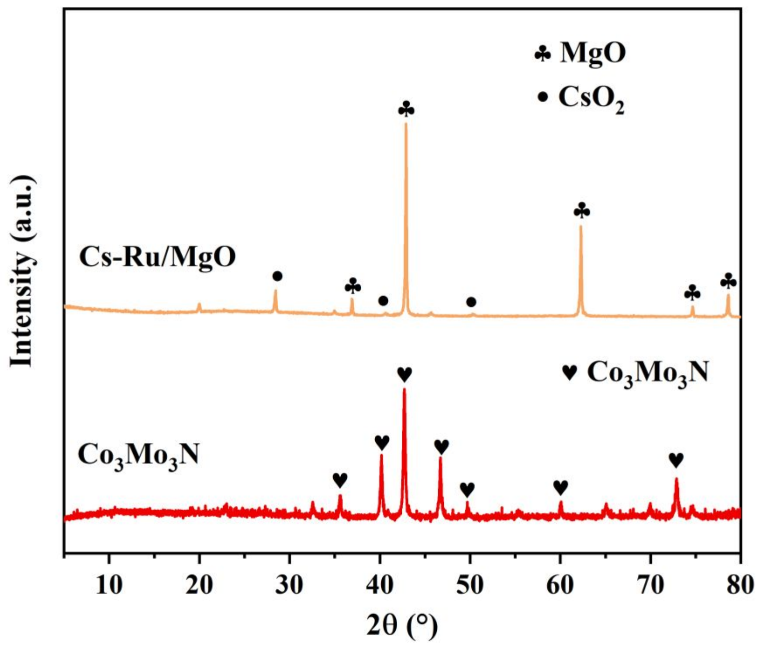

Figure S7. XRD patterns of Cs-5%Ru/MgO and Co_3Mo_3N.

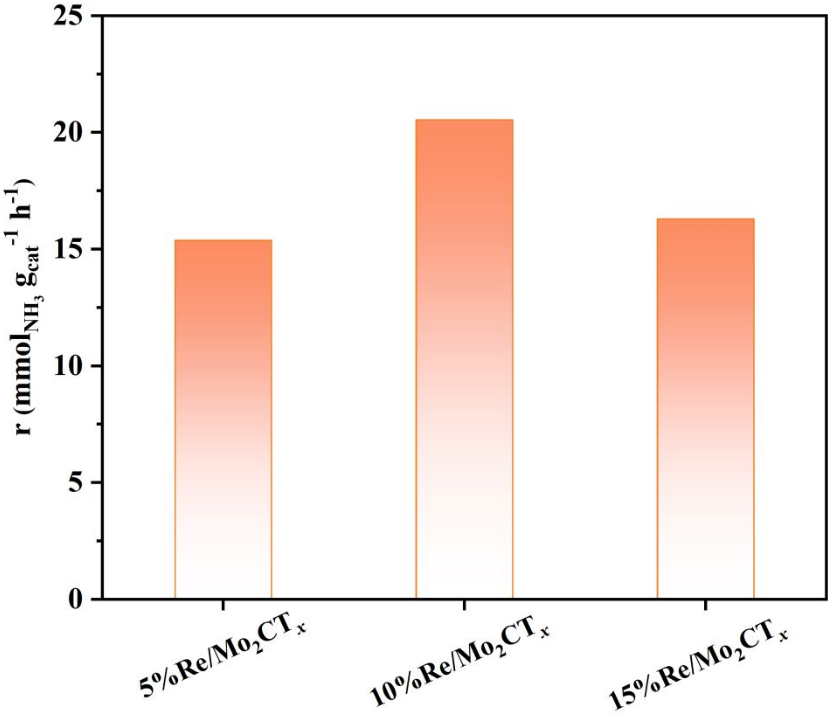

Figure S8. NH_3 synthesis rate over Re/Mo_2CT_x with different Re loadings.

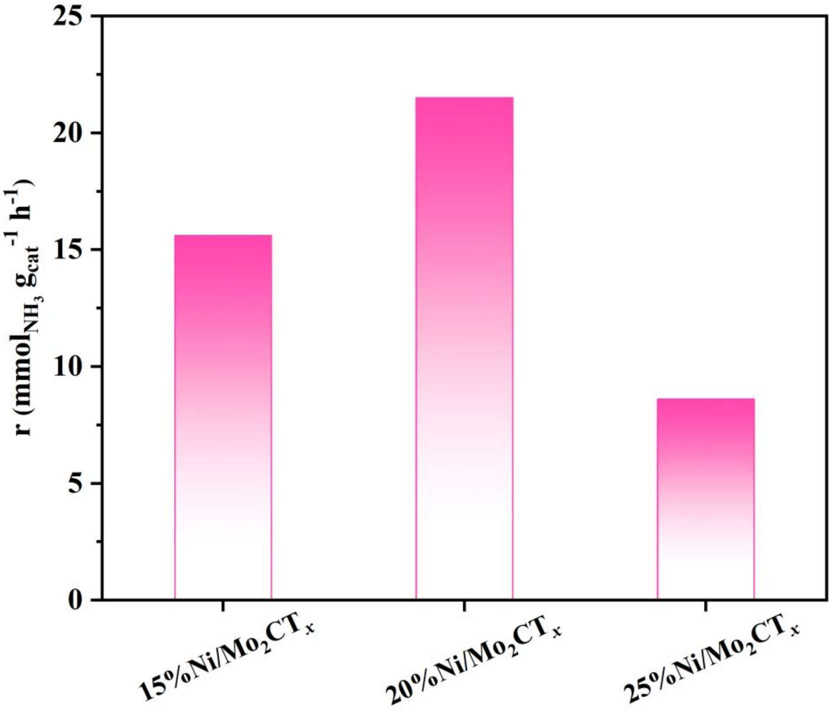

Figure S9. NH_3 synthesis rate over Ni/Mo_2CT_x with different Ni loadings.

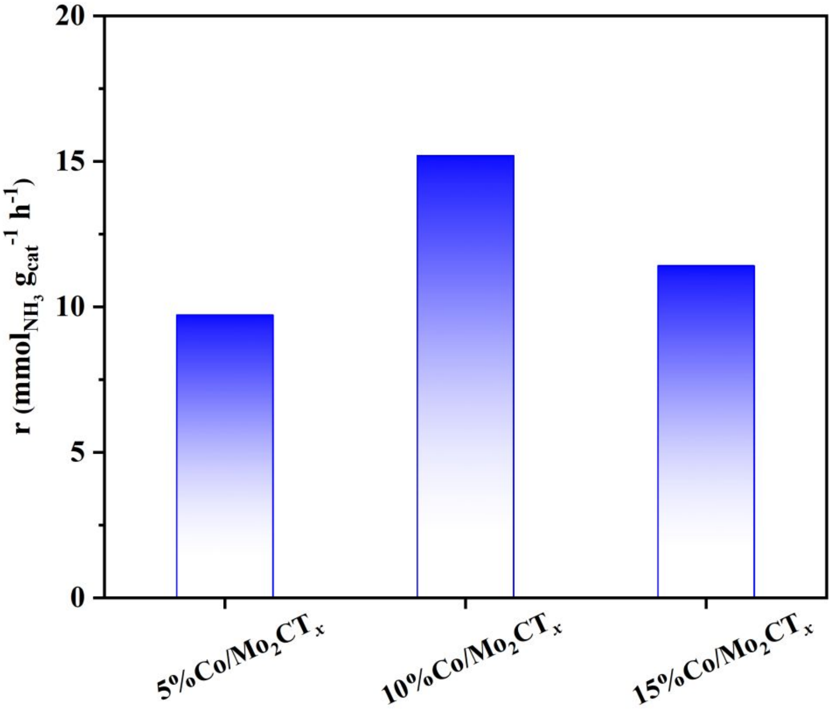

Figure S10. NH_3 synthesis rate over Co/Mo_2CT_x with different Co loadings.

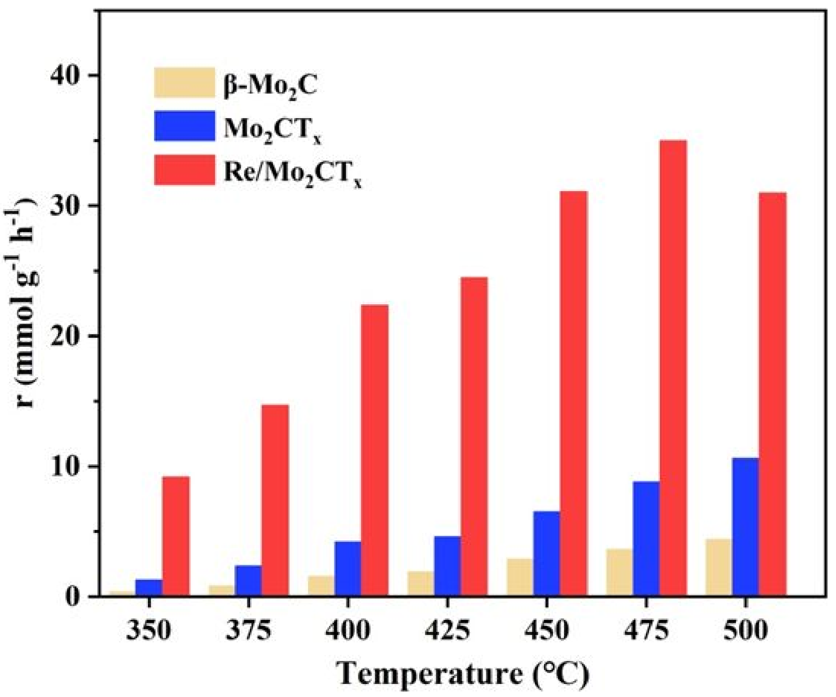

Figure S11. NH_3 synthesis rate versus reaction temperature over different catalysts at 1 MPa.

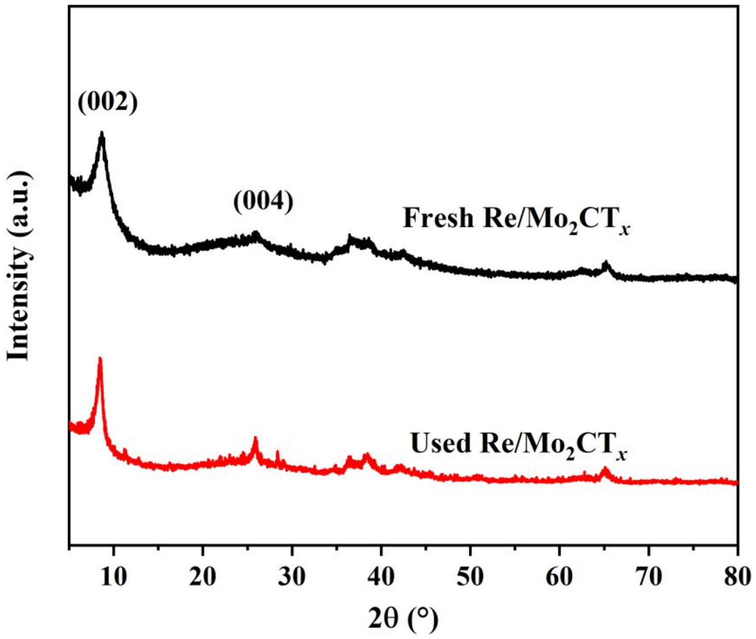

Figure S12. XRD patterns of fresh and used Re/Mo_2CT_x.

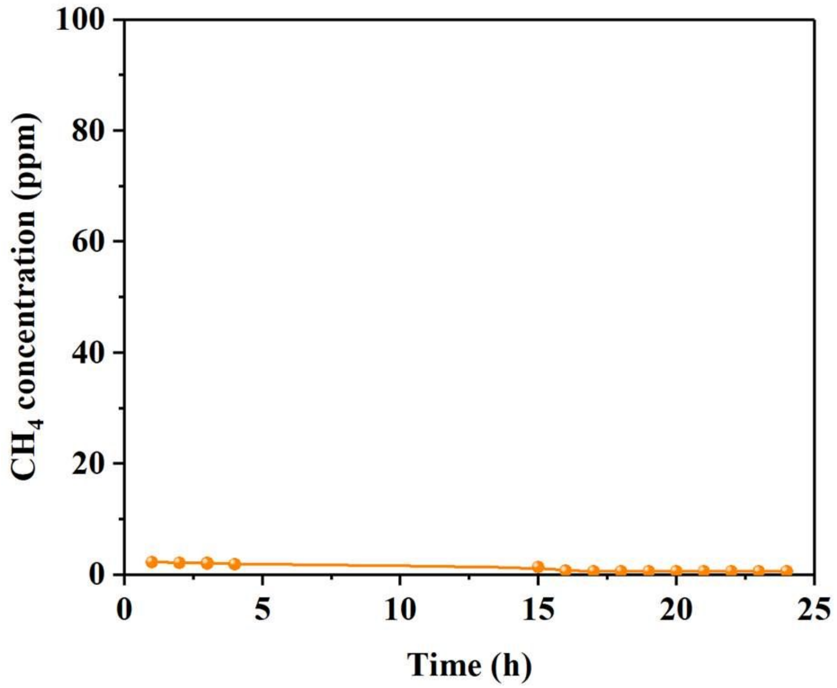

Figure S13. Outlet CH_4 concentration as a function of time during NH_3 synthesis over Re/Mo_2CT_x at 400 °C and 1 MPa, WHSV= 60 000 ml g^{-1} h^{-1}.

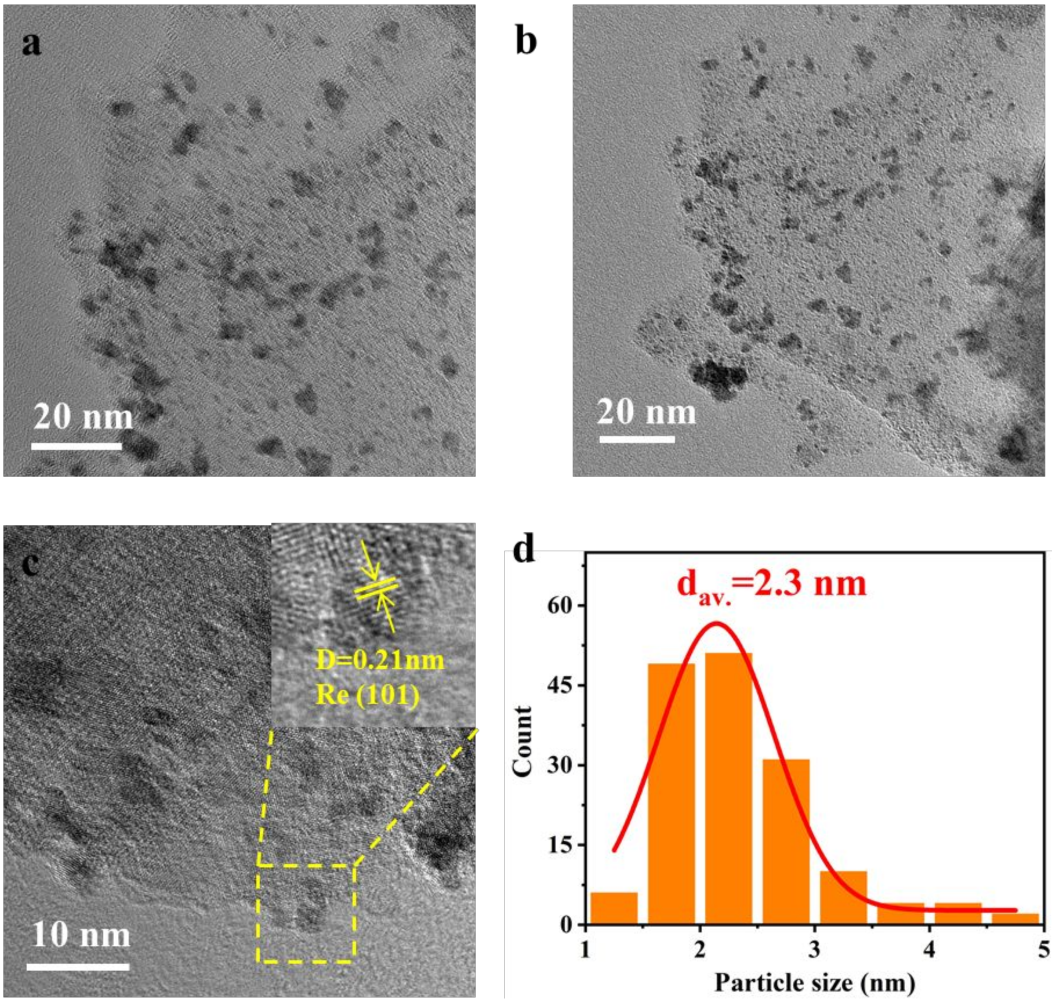

Figure S14. (a-c) HRTEM images and (d) corresponding Re particle size distribution of Re/Mo_2CT_x catalyst.

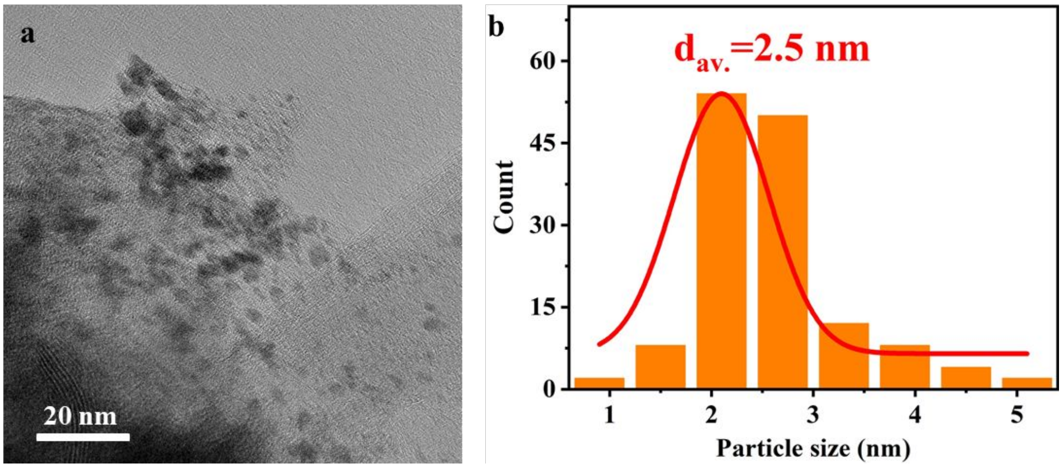

Figure S15. (a) HRTEM image and (b) corresponding Re particle size distribution of used Re/Mo_2CT_x catalyst.

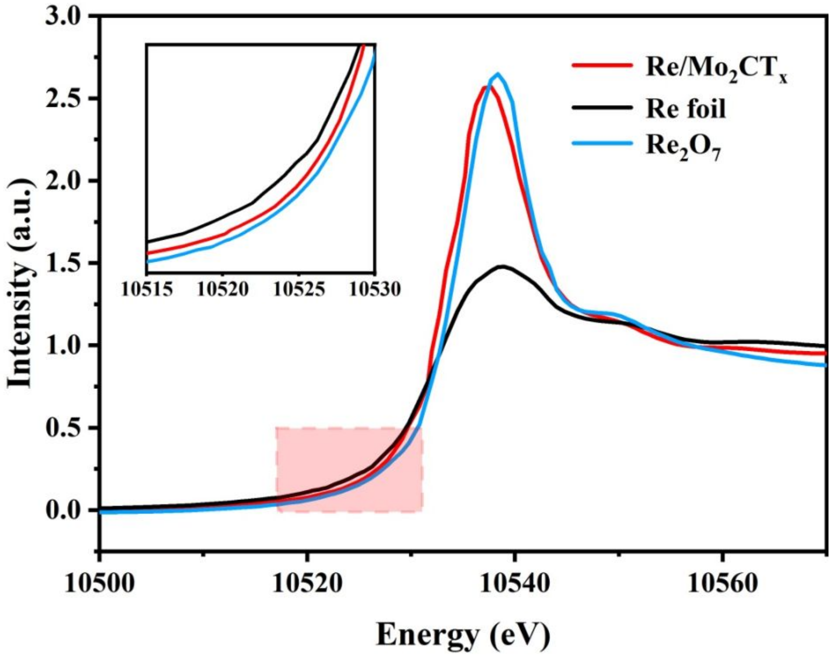

Figure S16. Normalized Re L-edge XANES of Re/Mo_2CT_x and reference samples.

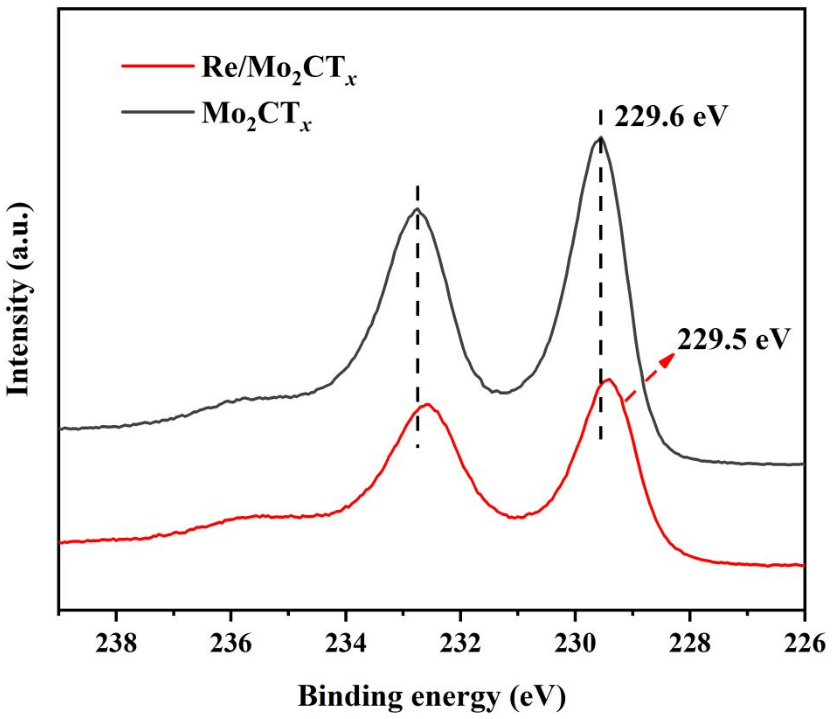

Figure S17. XPS spectra of Mo 3d of Mo_2CT_x and Re/Mo_2CT_x after H_2 treatment.

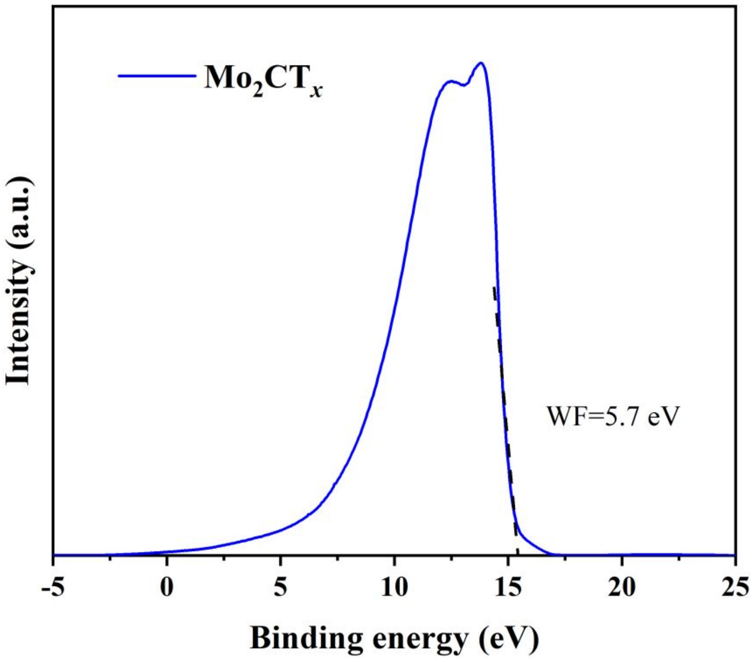

Figure S18. The UPS spectroscopy of Mo_2CT_x sample.

The work function of Mo_2CT_x determined by ultraviolet photoelectron spectroscopy (UPS) is 5.7 eV, which is higher that of metallic Re (4.3), Ni (4.6), and Co (5.0). Thus, the electron can transfer from TMs (Re, Ni, and Co) to Mo_2CT_x.

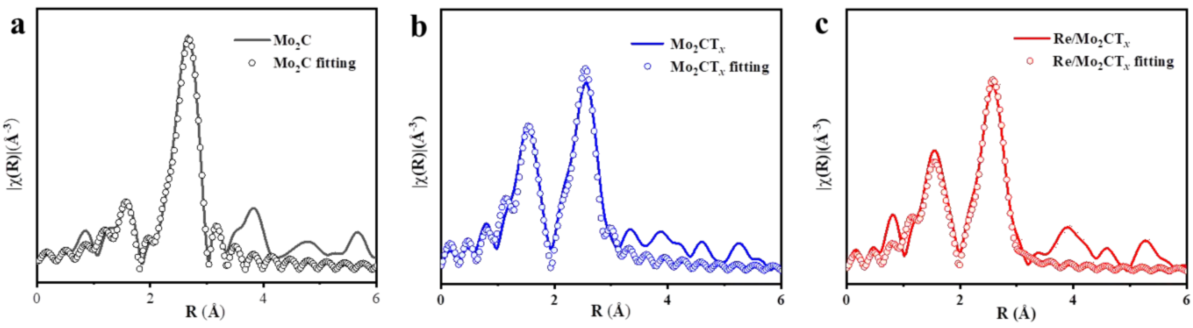

Figure S19. EXAFS spectra and the corresponding curve-fitting results of (a) β-Mo_2C, (b) Mo_2CT_x, (c) and Re/Mo_2CT_x.

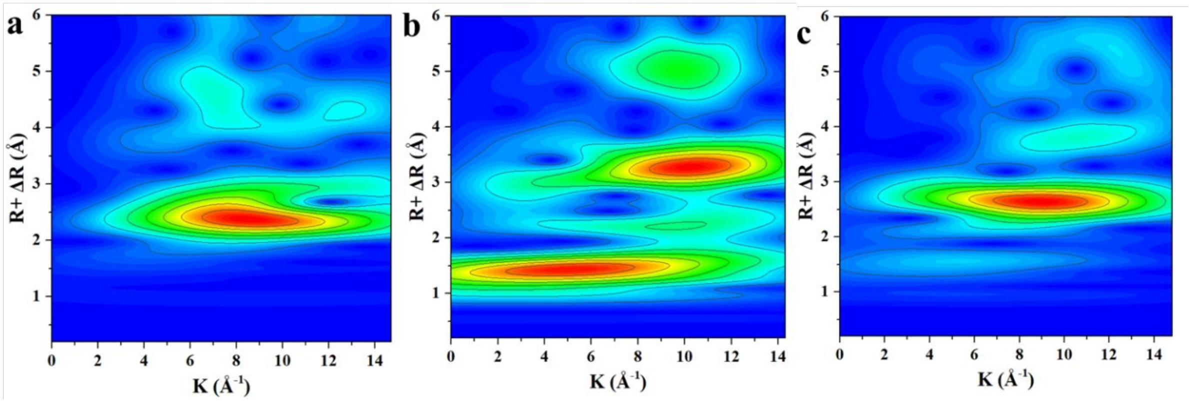

Figure S20. Wavelet transformation for k^2 weighted EXAFS signal of (a) Mo foil, (b) MoO_2, and (c) β-Mo_2C reference.

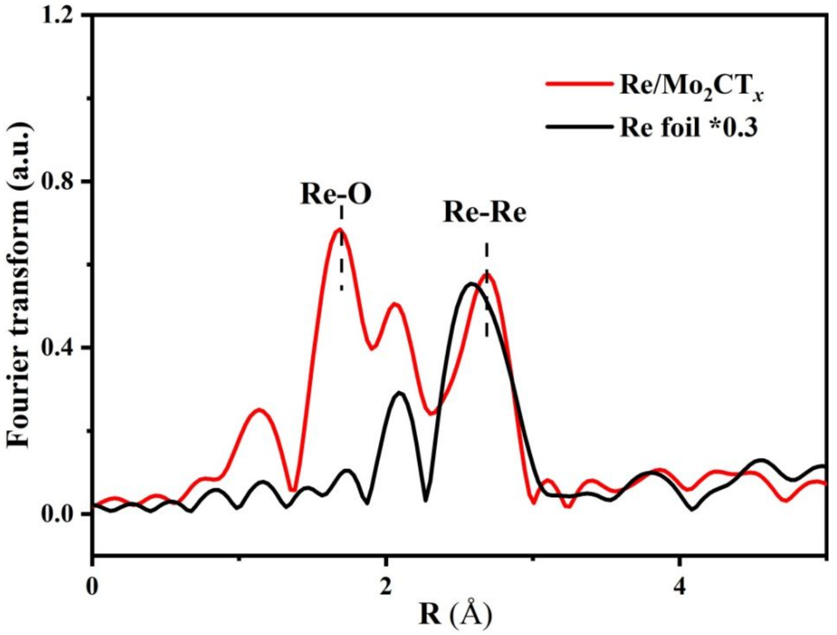

Figure S21. The k^2 weighted Fourier transform EXAFS spectra in *r*-space of Re/Mo_2CT_x and Re foil reference sample.

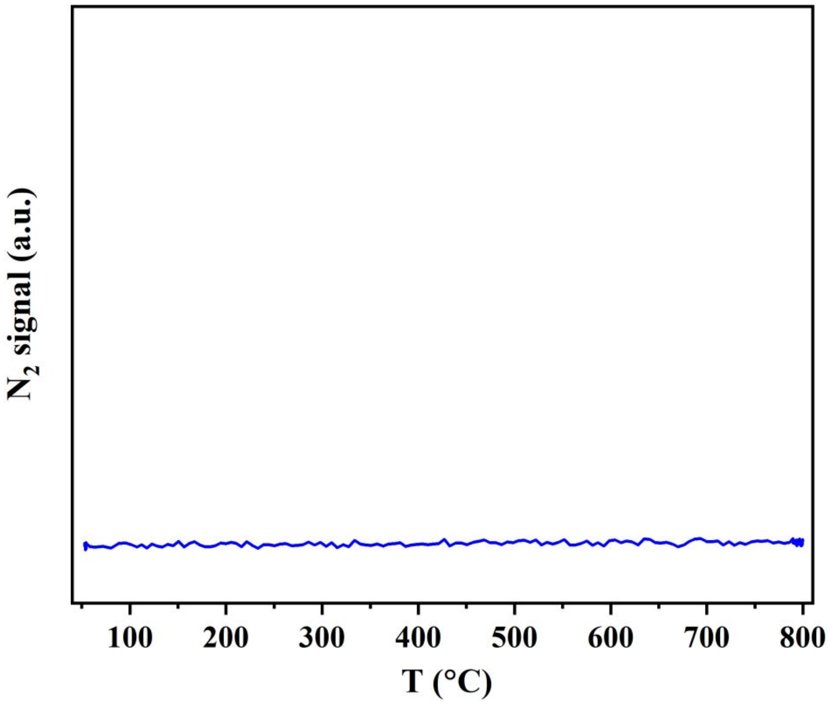

Figure S22. N_2-TPD-MS over pure Re metal.

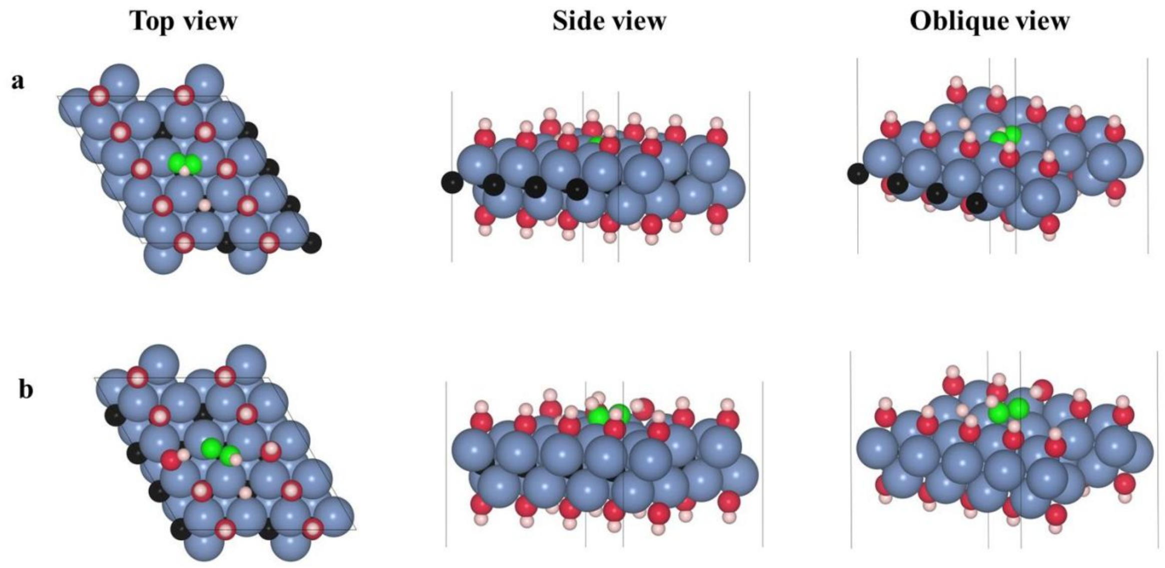

Figure S23. The dynamic adsorption of N_2 over the H-occupied surface of Mo_2COH, (a) the initial models and (b) the corresponding optimized models.

The H_2 is easily dissociated over the surface of Mo_2CT_x MXene, and the dissociated H atoms can occupy the hollow-C sites. When N_2 was placed beside the H atom, it can spontaneously occupy the site for H adsorption. It demonstrates the preferential adsorption of N_2 than H over Mo_2CT_x.

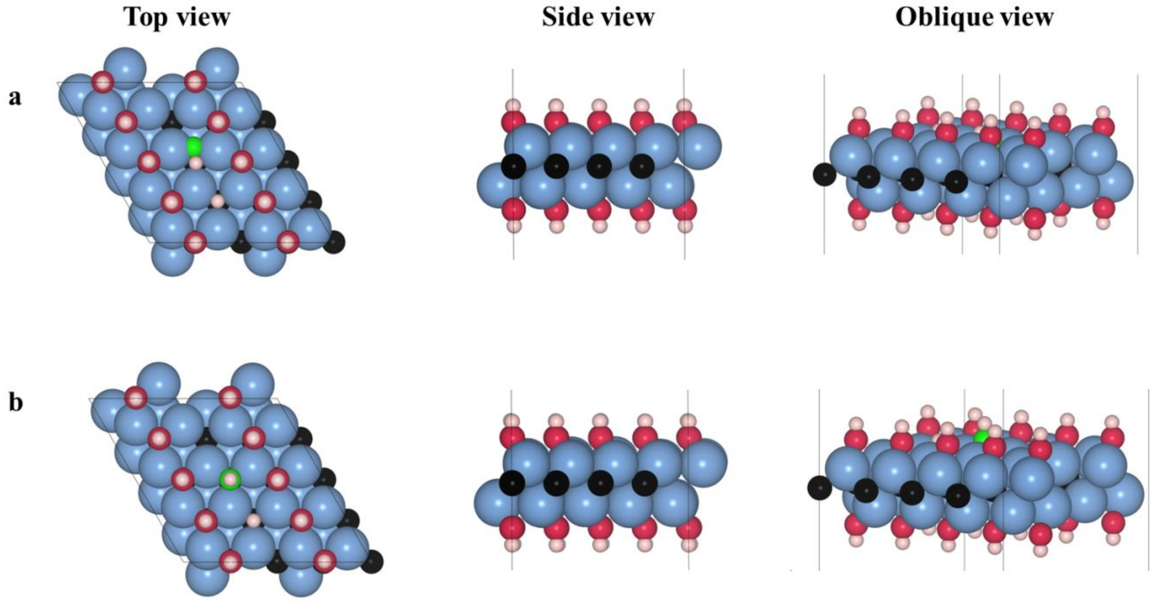

Figure S24. The dynamic adsorption of N atom over the H-occupied surface of Mo_2COH, (a) the initial models and (b) the corresponding optimized models.

The H_2 is easily dissociated over the surface of Mo_2CT_x MXene, and the dissociated H atoms can occupy the hollow-C sites. When N atom was placed beside the H atom, it can spontaneously occupy the site for H adsorption. It demonstrates the preferential adsorption of N than H over Mo_2CT_x.

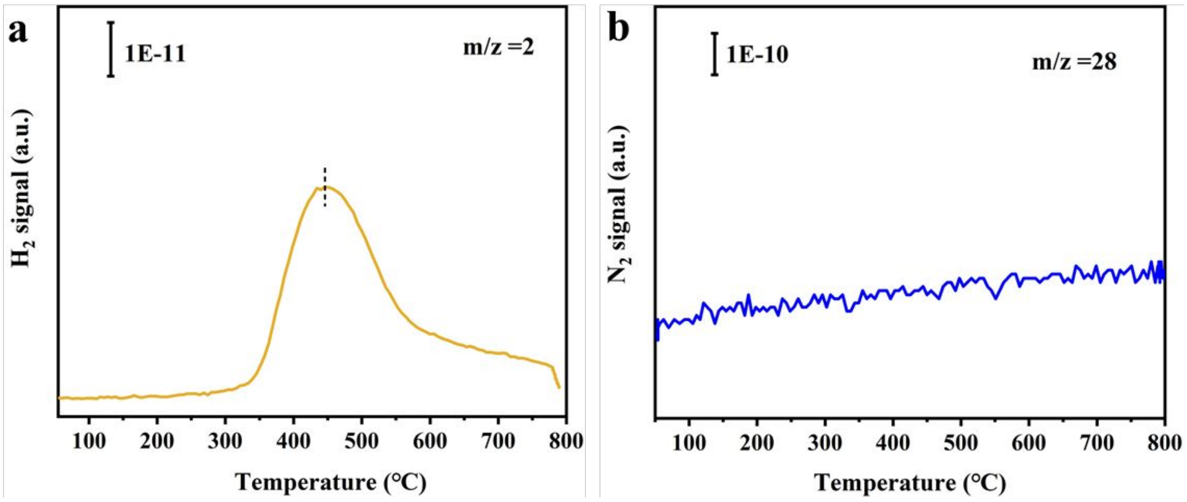

Figure S25. Signals of (a) H_2 and (b) N_2 desorption over Re metal after the co-adsorption of N_2 and H_2 gases.

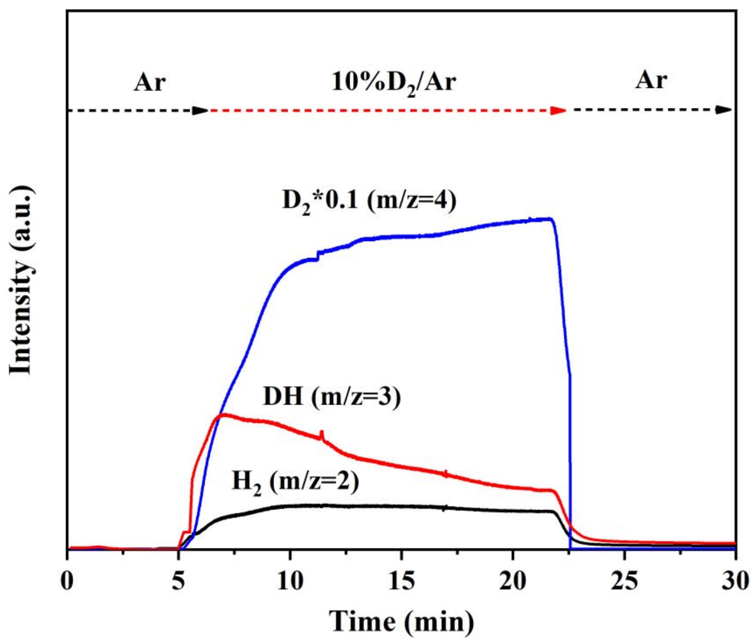

Figure S26. D_2 isotopic exchange experiment over Re/Mo_2CT_x at 400 °C.

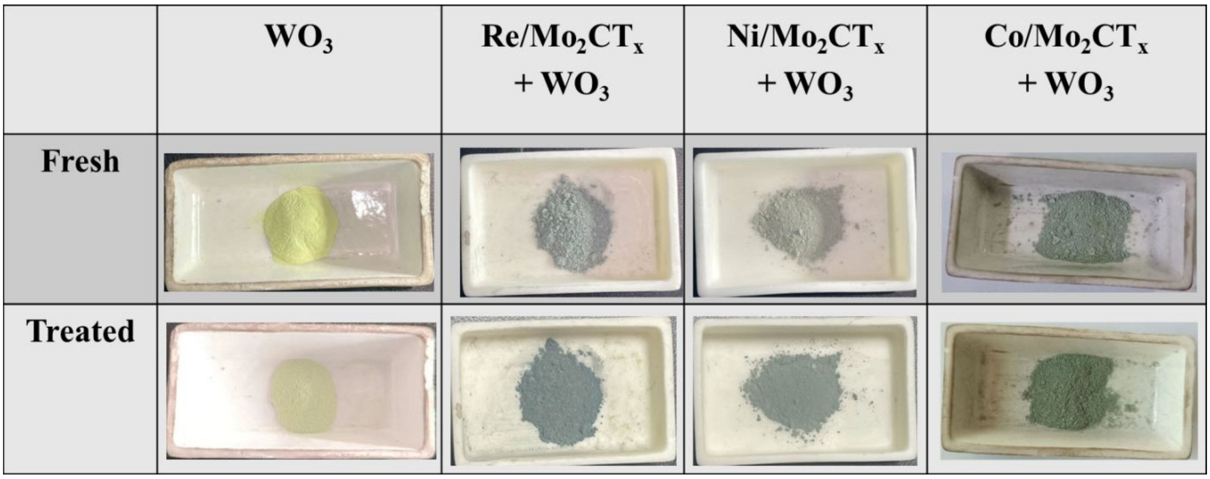

Figure S27. Photographs of WO_3, the mixture of WO_3 and NPMs/Mo_2CT_x catalysts before treatment and after hydrogen treatment at 250 °C for 10 min.

To further demonstrate the existence of H-spillover over Re/Mo_2CT_x, a color change experiment was conducted over the mixture of WO_3 and Re/Mo_2CT_x because the blue H_xWO_3 would form during hydrogen reduction of yellow WO_3. As depicted in Figure S27, hydrogen treatment of the mixture of WO_3 and Re/Mo_2CT_x leads to the color change from light yellow to dark blue. By comparison, no distinct color change is observed over WO_3. Similar phenomena are observed over Ni/Mo_2CT_x and Co/Mo_2CT_x catalysts. These results reveal that there exists the H-spillover over NPMs/Mo_2CT_x and the spillover hydrogen promotes the reduction of WO_3.

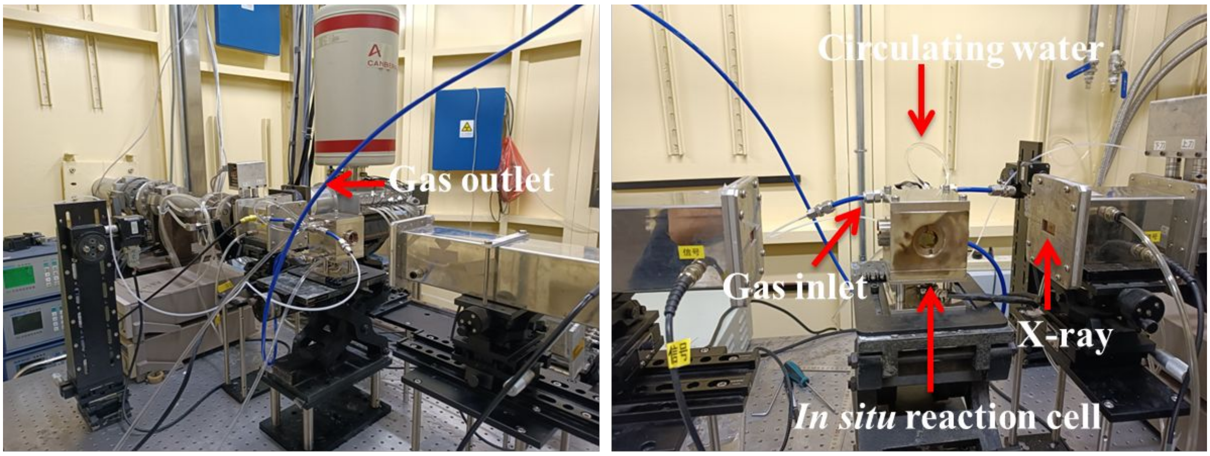

Figure S28. Pictures of equipment used for *in situ* XANES and EXAFS measurement.

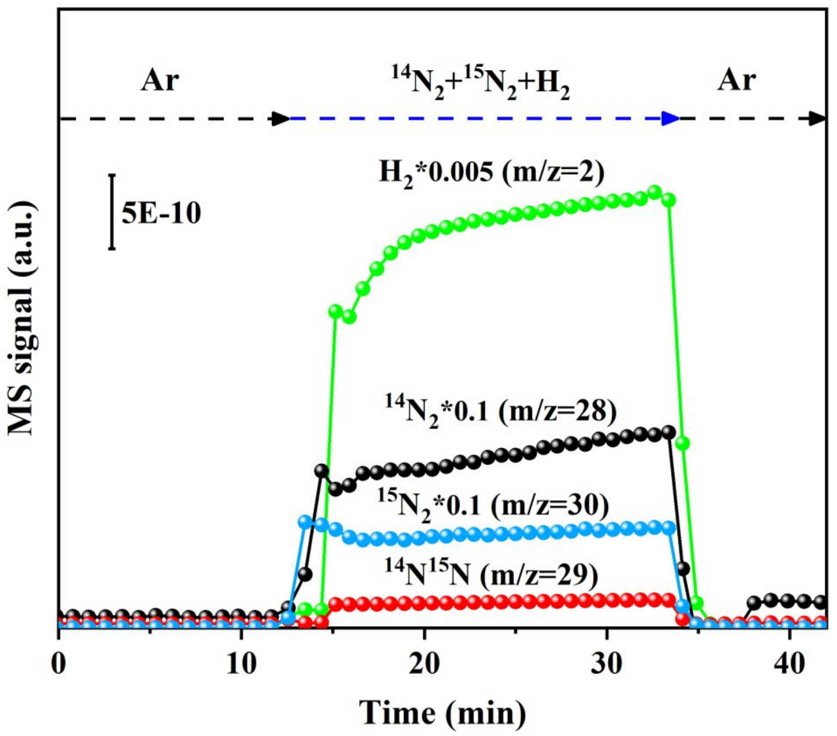

Figure S29. Mass signal over Mo_2CT_x after the introduction of $^{14}N_2$, $^{15}N_2$, and H_2 at 400 °C ($^{14}N_2/^{15}N_2/H_2$ volume ratio=2:1:6).

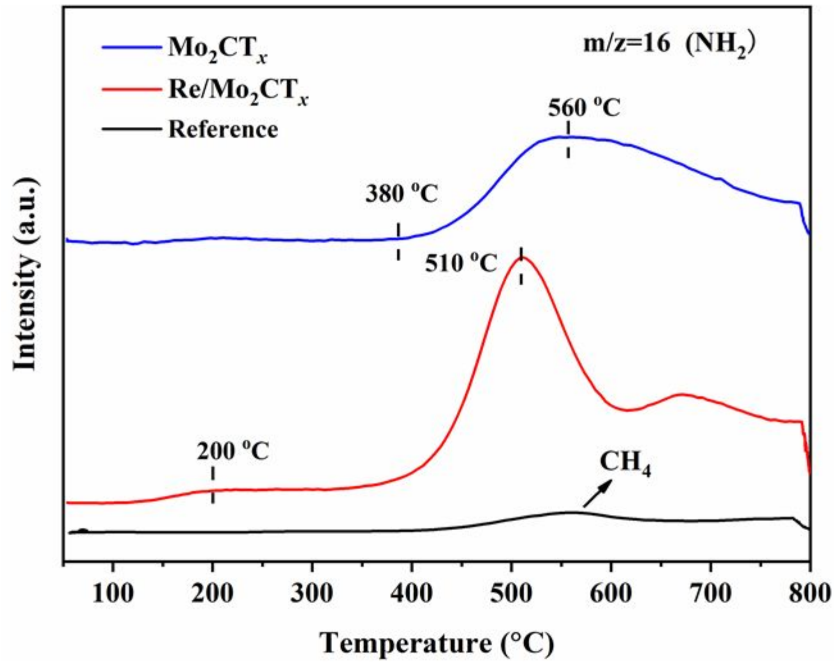

Figure S30. H_2-TPSR with an on-line MS detection over Mo_2CT_x and Re/Mo_2CT_x.

After Mo_2CT_x and Re/Mo_2CT_x were exposed to a N_2 atmosphere, H_2-temperature programmed surface reaction (H_2-TPSR) was performed to monitor NH_3 desorption signal. Meanwhile, Mo_2CT_x without exposure to N_2 atmosphere was used as a reference, in which the signal of m/z=16 is attributed to CH_4, originating from the methanation of the carbon species of Mo_2CT_x. It can be found that the signal of m/z=16 over the reference is much lower than that of Mo_2CT_x and Re/Mo_2CT_x. It demonstrates that the signal of m/z=16 over Mo_2CT_x and Re/Mo_2CT_x is mainly ascribed to NH_2 species.

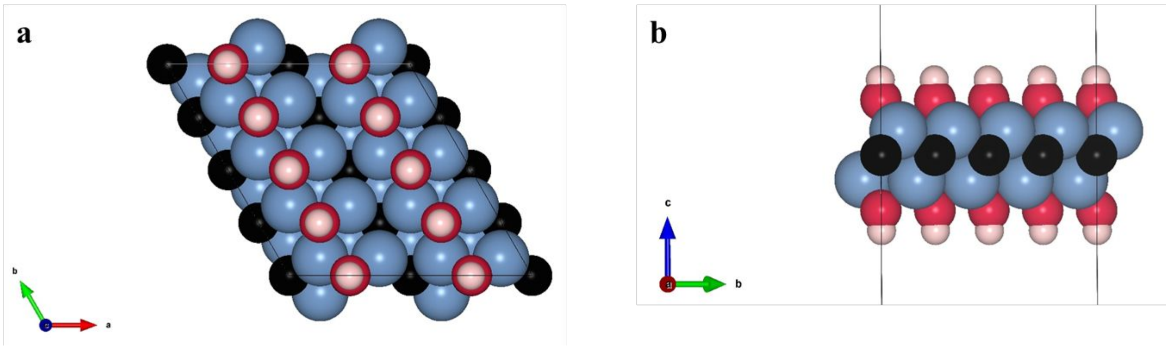

Figure S31. (a)Top view and (b) side view of Mo_2COH mode.

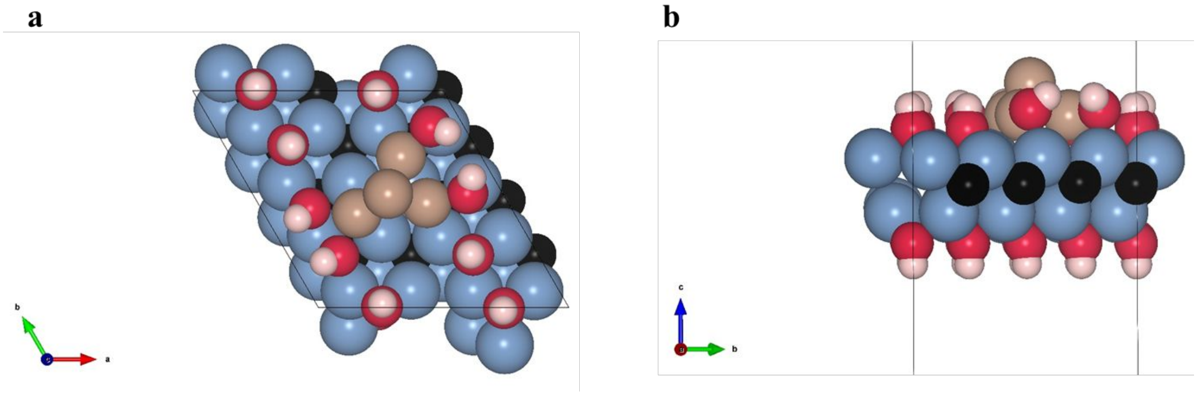

Figure S32. (a) Top view and (b) side view of Re/Mo_2COH mode.

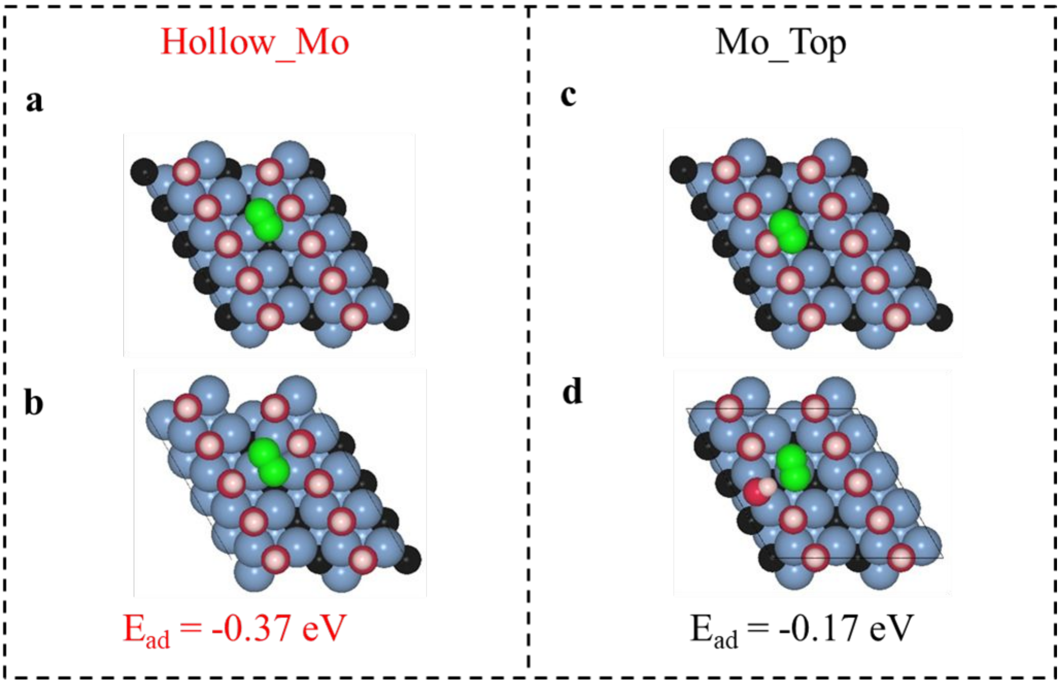

Figure S33. The constructed (a, c) and corresponding optimized (b, d) side-on adsorption configurations of N_2 on the surface of Mo_2COH. The configuration marked in red is the configuration used in the NEB calculation.

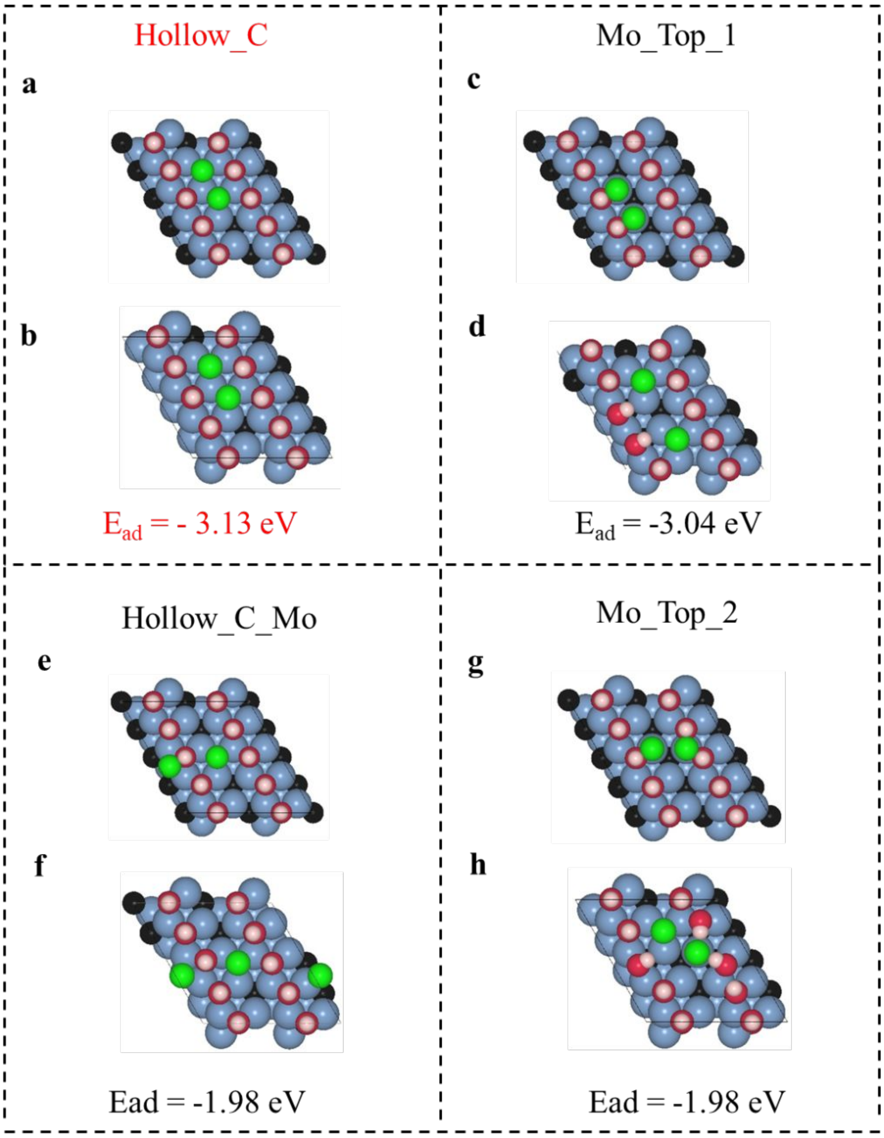

Figure S34. The constructed (a, c, e, g) and corresponding optimized (b, d, f, h) double-N configurations on the surface of Mo_2COH.

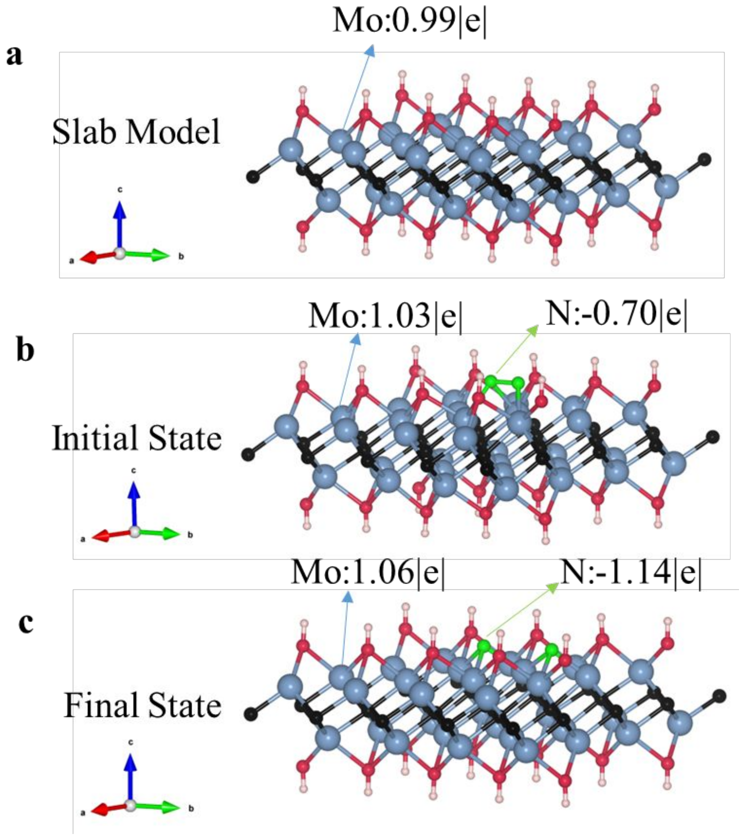

Figure S35. Bader charge analysis on Mo_2COH (a), Mo_2COH with N_2 adsorption (b) and Mo_2COH with 2N adsorption (c).

The charge amount marked in the figure is the average charge amount of single Mo atom and N atom.

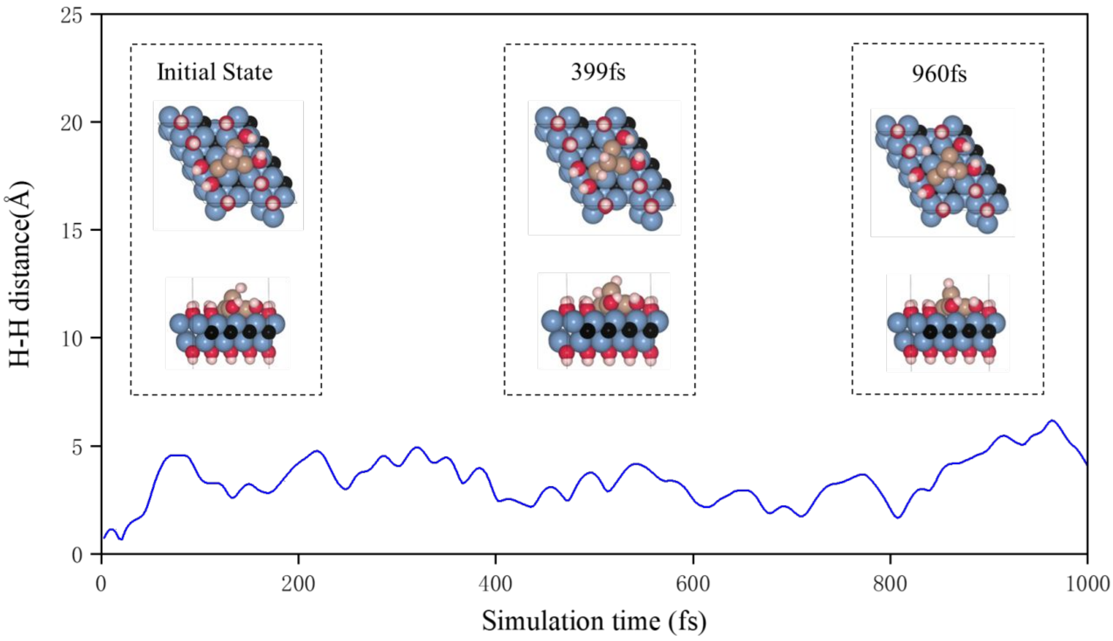

Figure S36. FPMD simulations of H_2 activation on Re/Mo_2COH.

In the FPMD calculation, Mo_2COH was fixed, and Re cluster and H_2 molecule were allowed to move during the FPMD calculation. Initially, H_2 molecule was placed near the metal Re surface. Firstly, H_2 molecule began to dissociate spontaneously into two H atoms. Then the H atom dynamically adsorbed and desorbed on the metal Re surface and Mo_2COH surface.

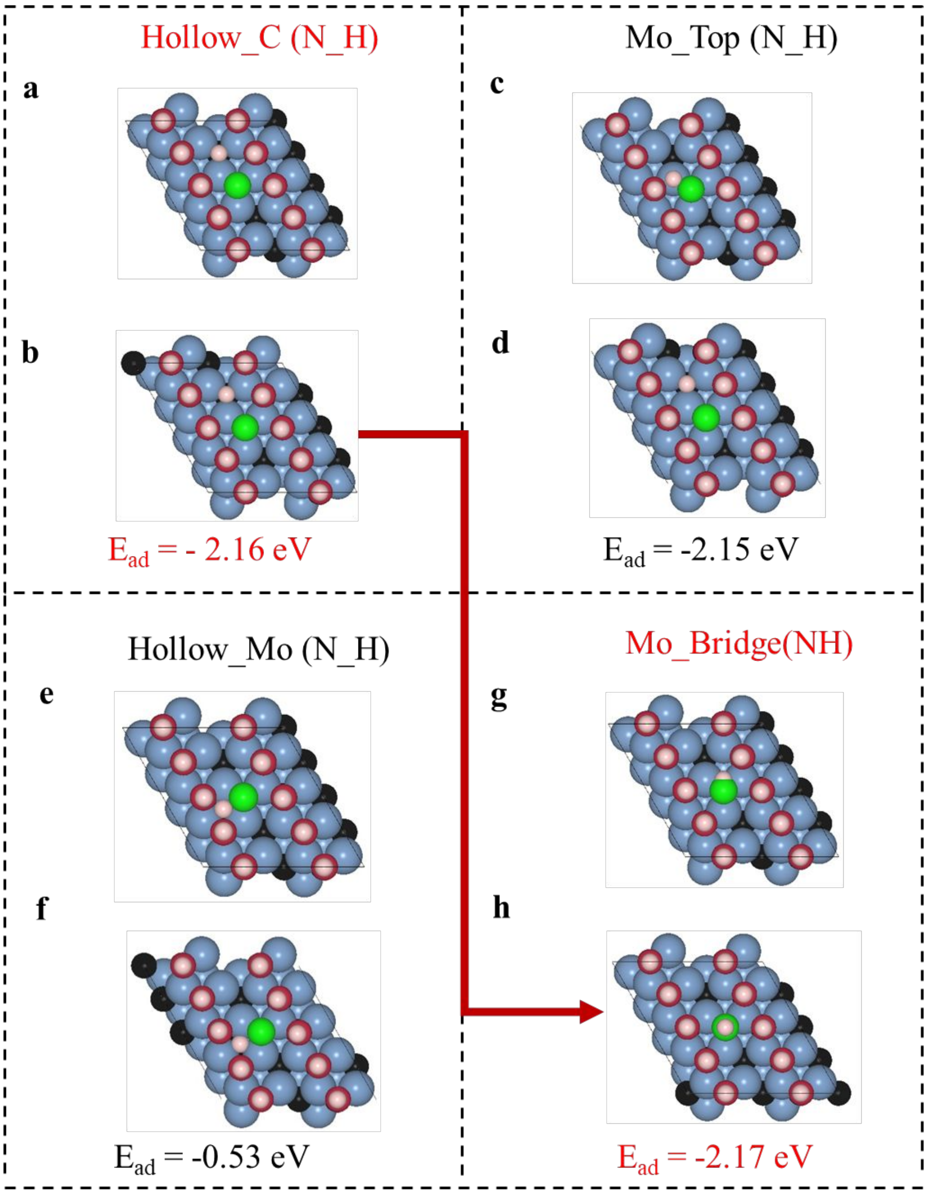

Figure S37. The constructed (a, c, e, g) and corresponding optimized (b, d, f, h) N_H and NH configurations on the surface of Mo_2COH. The arrow in the figure indicates the path of NH-formation.

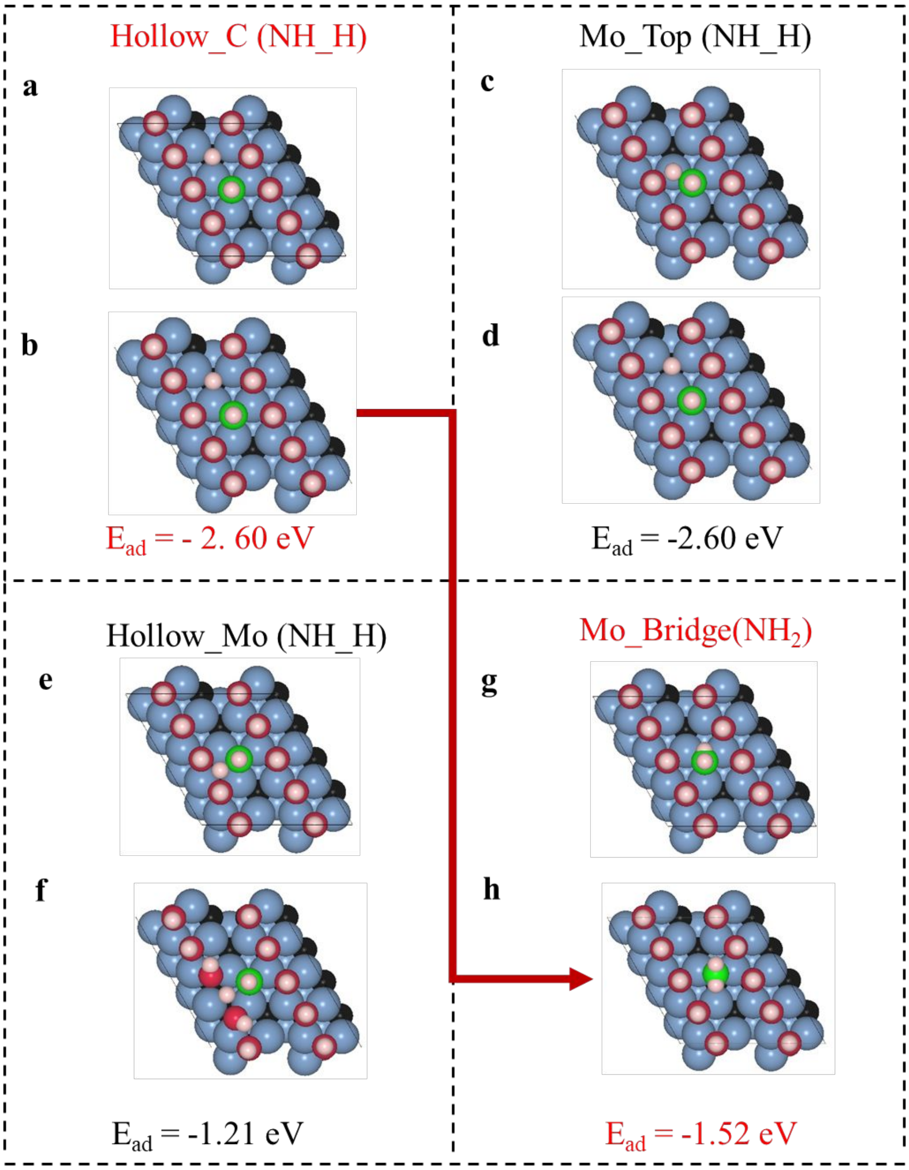

Figure S38. The constructed (a, c, e, g) and corresponding optimized (b, d, f, h) NH_H and NH_2 configurations on the surface of Mo_2COH. The arrow in the figure indicates the path of NH_2-formation.

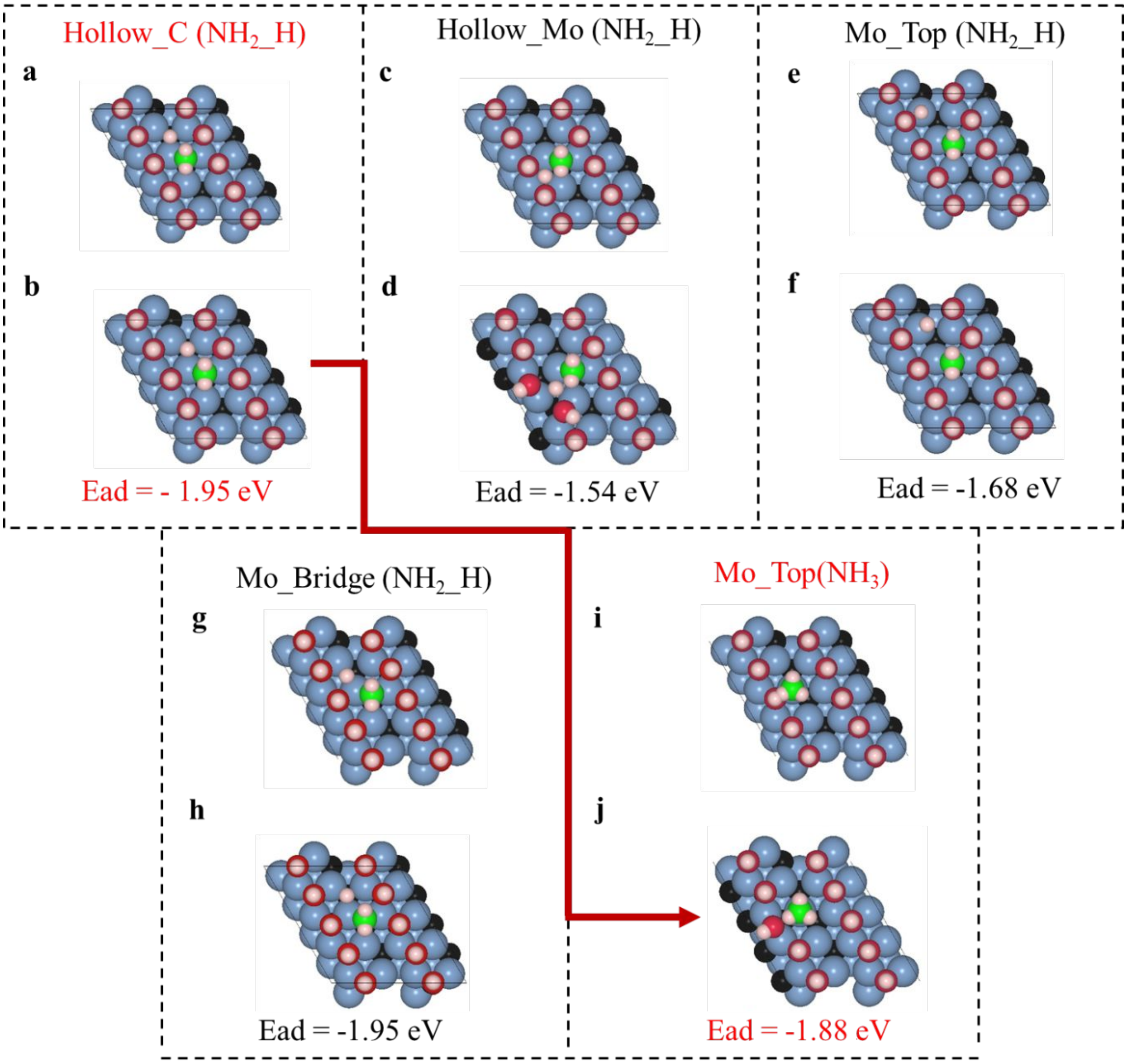

Figure S39. The constructed (a, c, e, g, i) and corresponding optimized (b, d, f, h, j) NH_2_H and NH_3 configurations on the surface of Mo_2COH. The arrow in the figure indicates the path of NH_3-formation.

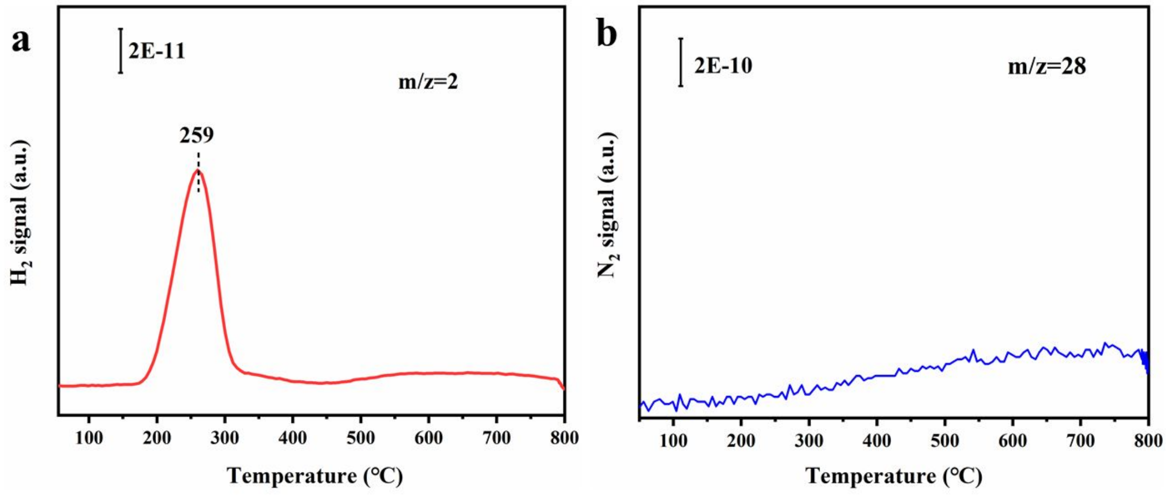

Figure S40. Signals of (a) H_2 and (b) N_2 desorption over Ni metal after the co-adsorption of N_2 and H_2 gases.

The amount of H_2 desorption is 0.037 mmol g^{-1}, while there is no N_2 desorption over Ni metal after exposure to 25%N_2-75%H_2 atmosphere.

3. Supporting Tables

Table S1. NH_3 synthesis performance over non-precious metal catalysts.

Sample	Metal content (wt.%)	Reaction conditions T (°C)	P (MPa)	WHSV (mL g^{-1} h^{-1})	NH_3 synthesis rate ($mmol_{NH3}$ g_{cat}^{-1} h^{-1})	E_a (kJ mol^{-1})	Ref.
Re/Mo_2CT_x	10	400	1.0	60 000	22.4	56.5	This work
Ni/Mo_2CT_x	20	400	1.0	60 000	21.5	65.2	This work
Ni/Mo_2CT_x	20	400	0.9	36 000	16.3	--	This work
Co-Mo/CeO_2	--	400	0.9	--	2.84	61.0	3
β-Mo_2C	--	400	3.1	9 000	4.3	53.0	4
Co/Mo_2C	22	520	0.5	9 000	1.0	55.8	5
MoH_x/SiO_2	2	400	1.0	12 000	0.12	--	6
MoN_x/ZSM-5	2.2	400	2.0	9 000	5.7	105	7
Cs-Re/Al_2O_3	20.0	350	1.1	9 000	1.7	--	8
Ni/LaN	12.5	400	0.9	36 000	15.5	57.5	9
Co-NC	3.8	400	1.0	60 000	4.34	50.0	10
Co-LiH	10.0	350	1.0	60 000	11.5	52.1	11
BaH_2-Co/CNTs	10.0	400	1.0	60 000	21.0	58.0	12
Co/C12A7:e^-	2.6	400	0.9	36 000	3.9	49.5	13
LaCoSi	26	400	0.9	36 000	5.0	42.0	14

Table S2. EXAFS data fitting results of the as-synthesized catalysts.

Samples	Shell	CN[a]	R/Å[b]	σ^2×(10^{-2})/Å^2 [c]	ΔE_0/eV[d]	R-factor (%)
β-Mo_2C	Mo-C	3*[e]	2.09	0.1	-0.6	1.1
	Mo-C-Mo1	6*[e]	2.92	0.1	-0.6	1.1
	Mo-C-Mo2	6*[e]	3.2	0.1	-0.6	1.1
Mo_2CT_x	Mo-C	6.6±0.5	2.10	0.1	-6.1	1.7
	Mo-C-Mo1	1.2±0.4	2.74	0.2	-6.1	1.7
	Mo-C-Mo2	5.5±0.4	2.88	0.1	-6.1	1.7
Re/Mo_2CT_x	Mo-C	5.6±0.6	2.13	0.2	-2.3	1.8
	Mo-C-Mo1	1.0±0.3	2.74	0.1	-2.3	1.8
	Mo-C-Mo2	7.1±0.3	2.89	0.2	-2.3	1.8

[a]CN, coordination number; [b]R, bonding distance; [c]σ^2, the Debye−Waller factor; [d]ΔE_0, inner potential shift, [e]Number with an asterisk indicates fixed coordination number (*N*) according to the crystal structure; The accuracies of the above parameters are estimated as CN, ±20%; R, ±1%; σ^2, ± 20%; ΔE_0 , ±20%. The data range used for data fitting in k-space (Δk) and R-space (ΔR) are 3.0–12.8 Å^{-1} and 1.0–3.0 Å, respectively.

Table S3. The chemical composition of Mo and Re elements over Re/Mo_2CT_x acquired from quasi *in situ* XPS experiments.

Reaction atmosphere	Mo^{4+} (%)	Mo^{6+} (%)	Mo^{4+}/Mo^{6+} (%)	Re^0 (%)	Re^{2+} (%)	Re^0/Re^{2+} (%)
Fresh	65	35	1.9	45.1	54.9	0.82
10%H_2/Ar	88	12	7.3	51.0	49.0	1.03
2.5%N_2/Ar	78	22	3.5	50.5	49.5	1.02
2.5%N_2+7.5%H_2/Ar	81	19	4.3	51.2	48.8	1.05

Table S4. The amount of N_2 and H_2 desorption over as-synthesized catalysts.

Catalyst	Metal loading (wt.%)	N_2 desorption[a] (mmol g_{cat}^{-1})	N_2 desorption[b] (mmol g_{cat}^{-1})	H_2 desorption[b] (mmol g_{cat}^{-1})
Re	100	0	0	0.06
Mo_2CT_x	0	0.60	0.55	0.33
Re/Mo_2CT_x	10	0.62	0.51	0.71
Ni/Mo_2CT_x	20	--	0.41	0.72
Co/Mo_2CT_x	10	--	0.43	0.65

[a]Amount of N_2 desorption determined by N_2-TPD.

[b]Amount of N_2 and H_2 desorption measured after N_2 and H_2 co-adsorption over catalysts.

Table S5. EXAFS data fitting results of Re/Mo_2CT_x catalyst.

Treatment conditions	Shell	CN[a]	R/Å[b]	$\sigma^2 \times (10^{-2})$/Å^2 [c]	ΔE_0/eV[d]	R-factor (%)
H_2 reduction for 45 min	Mo-C	5.5	2.13	0.5	-2.1	0.9
	Mo-C-Mo1	1.0	2.75	0.3	-2.1	0.9
	Mo-C-Mo2	7.0	2.88	0.3	-2.1	0.9
N_2/H_2 for 15 min	Mo-C(N)	5.6	2.14	0.4	4.6	0.9
	Mo-C-Mo1	1.0	2.77	0.3	0.2	0.9
	Mo-C-Mo2	7.0	2.88	0.3	2.2	0.9
N_2/H_2 for 30 min	Mo-C(N)	6.2	2.13	0.5	-3.0	0.6
	Mo-C-Mo1	1.0	2.76	0.3	-3.0	0.6
	Mo-C-Mo2	7.1	2.89	0.3	-3.0	0.6
N_2/H_2 for 45 min	Mo-C(N)	6.3	2.13	0.5	-2.3	0.8
	Mo-C-Mo1	1.0	2.76	0.3	-2.3	0.8
	Mo-C-Mo2	7.0	2.89	0.3	-2.3	0.8

[a]CN, coordination number; [b]R, bonding distance; [c]σ^2, the Debye−Waller factor; [d]ΔE_0, inner potential shift, [e]Number with an asterisk indicates fixed coordination number (*N*) according to the crystal structure; The accuracies of the above parameters are estimated as CN, ±20%; R, ±1%; σ^2, ± 20%; ΔE_0 , ±20%. The data range used for data fitting in k-space (Δk) and R-space (ΔR) are 3.0–12.8 Å^{-1} and 1.0–3.0 Å, respectively.

Table S6. The frequencies for transition state of H spillover from Re to Mo_2COH.

No.	Elementary Step	TS Barrier/ eV	Frequency/cm^{-1}
1	H spillover	0.37	1305.66, 697.76, **366.40**[i]

Table S7. The frequencies for transition states of each elementary step on Mo_2COH.

No.	Elementary Step	TS Barrier/ eV	Frequency/cm^{-1}
1	N_2*=2N*	0.23	590.95, 581.41, 439.77, 427.69, 218.59, **513.43**[i]
2	N*+H*=NH*	1.09	1590.50, 697.04, 552.04，455.43，346.07，**1222.30**[i]
3	NH*+H*=NH_2*	1.48	3390.04, 1659.76, 978.20, 685.47, 586.69, 519.36, 415.24, 286.21,**1109.50**[i]
4	NH_2*+H*=NH_3*	1.09	3508.03, 3367.09, 1464.40, 1068.98, 745.60, 684.69, 483.92, 379.32, 300.72, 211.15, 155.64,**1337.35**[i]

* Mo_2COH slab; i: A imaginary frequency of transition state.

References

1. Aika K., *et al.* Support and promoter effect of ruthenium catalyst. III. Kinetics of ammonia synthesis over various Ru catalysts. *Appl. Catal.* **28**, 57-68 (1986).
2. Iriawan H., *et al.* Methods for nitrogen activation by reduction and oxidation. *Nat. Rev. Meth. Primers* **1**, 56 (2021).
3. Yuki Tsuji, *et al.* Ammonia synthesis over Co-Mo alloy nanoparticle catalyst prepared by sodium naphthalenide-driven reduction. *Chem. Commun.* **100**, 14369–14372 (2016).
4. Kojima R., Aika K. Molybdenum nitride and carbide catalysts for ammonia synthesis. *Appl. Catal. A-Gen.* **219**, 141–147 (2001).
5. Roy P. K., Kumar S. Strong interfacial electronic interaction in the transition-Metal/Mo_2C catalyst for enhanced ammonia synthesis at ambient pressure: shift of the rate determining step. *ACS Appl. Energy Mater.* **3**, 7167–7179 (2020).
6. Azofra L. M., *et al.* Single-site molybdenum on solid support materials for catalytic hydrogenation of N_2-into-NH_3. *Angew. Chem. Int. Ed.* **57**, 15812–15816 (2018).
7. Liu N., *et al.* Catalytic ammonia synthesis over Mo nitride/ZSM-5. *ChemCatChem* **2**, 167–174 (2010).
8. Kojima R., Enomoto H., Muhler M., Aika K.-i. Cesium-promoted rhenium catalysts supported on alumina for ammonia synthesis. *Appl. Catal. A-Gen.* **246**, 311–322 (2003).
9. Ye T. N., *et al.* Vacancy-enabled N_2 activation for ammonia synthesis on an Ni-loaded catalyst. *Nature* **583**, 391–395 (2020).
10. Wang X., *et al.* Insight into dynamic and steady-state active sites for nitrogen activation to ammonia by cobalt-based catalyst. *Nat. Commun.* **11**, 653 (2020).
11. Wang P., *et al.* Breaking scaling relations to achieve low-temperature ammonia synthesis through LiH-mediated nitrogen transfer and hydrogenation. *Nat. Chem.* **9**, 64–70 (2017).

12. Gao W., et al. Barium hydride-mediated nitrogen transfer and hydrogenation for ammonia synthesis: a case study of cobalt. ACS Catal. 7, 3654-3661 (2017).

13. Inoue Y., *et al.* Direct activation of cobalt catalyst by $12CaO{\cdot}7Al_2O_3$ electride for ammonia synthesis. *ACS Catal.* **9**, 1670–1679 (2019).

14. Gong Y., *et al.* Ternary intermetallic LaCoSi as a catalyst for N_2 activation. *Nat. Catal.* **1**, 178–185 (2018).
